# Topology of pain networks in patients with temporomandibular disorder and pain-free controls with and without concurrent experimental pain: A pilot study

**DOI:** 10.3389/fpain.2022.966398

**Published:** 2022-10-17

**Authors:** Jeremy L. Smith, Jason W. Allen, Candace C. Fleischer, Daniel E. Harper

**Affiliations:** ^1^ Department of Radiology and Imaging Sciences, Emory University School of Medicine, Atlanta, GA, United States; ^2^ Department of Neurology, Emory University School of Medicine, Atlanta, GA, United States; ^3^ Department of Biomedical Engineering, Georgia Institute of Technology and Emory University, Atlanta, GA, United States; ^4^ Department of Anesthesiology, Emory University School of Medicine, Atlanta, GA, United States; ^5^ Department of Anesthesiology, University of Michigan, Ann Arbor, MI, United States

**Keywords:** temporomandibular disorder (TMD), resting state—fMRI, graph theory, chronic pain, quantitative sensory testing (QST)

## Abstract

Temporomandibular disorders (TMD) involve chronic pain in the masticatory muscles and jaw joints, but the mechanisms underlying the pain are heterogenous and vary across individuals. In some cases, structural, functional, and metabolic changes in the brain may underlie the condition. In the present study, we evaluated the functional connectivity between 86 regions of interest (ROIs), which were chosen based on previously reported neuroimaging studies of pain and differences in brain morphology identified in an initial surface-based morphometry analysis. Our main objectives were to investigate the topology of the network formed by these ROIs and how it differs between individuals with TMD and chronic pain (*n* = 16) and pain-free control participants (*n* = 12). In addition to a true resting state functional connectivity scan, we also measured functional connectivity during a 6-min application of a noxious cuff stimulus applied to the left leg. Our principal finding is individuals with TMD exhibit more suprathreshold correlations (higher nodal degree) among all ROIs but fewer “hub” nodes (i.e., decreased betweenness centrality) across conditions and across all pain pathways. These results suggest is this pain-related network of nodes may be “over-wired” in individuals with TMD and chronic pain compared to controls, both at rest and during experimental pain.

## Introduction

Chronic temporomandibular disorders (TMD) involve persistent pain in the masticatory muscles and jaw joints and in many cases dysfunction of the jaw. Peripheral nociceptive pain elicited by inflammation or injury in the temporomandibular joint and surrounding muscles and tendons can contribute to clinical pain in the area, but central nervous system changes in at least a subset of individuals with TMD can also lead to centralized, or nociplastic, pain (1). Decades of research in a variety of regional pain conditions including TMD, chronic pelvic pain, irritable bowel syndrome, migraine, vulvodynia, and others, as well as more widespread pain as is observed in fibromyalgia, have shown that chronic pain is associated with differences in brain structure, function, and neurochemistry, compared to chronic pain-free individuals (2). In addition, many patients with chronic pain exhibit alterations in experimentally evoked noxious stimulus processing, including hyperalgesia, allodynia, and impaired ability to reduce ascending nociceptive signals through endogenous activation of the descending pain modulatory system (3, 4). Thus, brain neuroimaging of TMD patients who are undergoing experimental pain could reveal underlying functional differences in nociceptive processing.

Central nociceptive functions are subserved by cortical-subcortical pathways, including the trigemino-thalamocortical (CN V-TC) and periaqueductal-rostral ventromedial medulla (PAG-RVM) pathways, as well as distributed cerebral networks, such as the sensory/discriminatory lateral and affective/cognitive medial pain systems (LPS and MPS, respectively) (5–8). These nociceptive systems, however, do not function in isolation. They are closely associated with other cortical and subcortical networks which primarily subserve affective, cognitive, motivational, and integrative functions, but are jointly activated with the above nociceptive systems and pathways during pain sensation. For example, a corpus of brain areas, originally described as a “pain neuromatrix,” are involved in the nociceptive (posterior insular, middle cingulate, and medial parietal opercular cortices) or perceptual aspects of pain (middle and anterior insular, prefrontal, posterior parietal, supplementary motor, and anterior cingulate cortices, hippocampus, striatum, and cerebellum) (5, 9–16); however, it should be noted that most of these areas are not specific to pain processing. Additionally, alterations in grey matter volume, neurochemistry, and connectivity within the default-mode network (DMN), which functions in monitoring and processing internal states (17–22), are correlated with pain sensitivity, severity, duration, catastrophizing, and rumination (16, 23–26). Finally, several regions which mediate body perception, learning and motivational functions, and pain anticipation, perception, and empathy, are often omitted from even the broadly-defined pain neuromatrix. This is due, in part, to the absence of a consensus definition of what areas constitute this neuromatrix and a lack of specificity of these regions for processing nociception. These regions include the fusiform cortex, a component of the dopaminergic reward system which is not only implicated in body and face recognition but also in pain anticipation and perception (27–32); other components of the dopaminergic reward system, such as the ventral pallidum and planum polare (33–35); the supramarginal and angular gyri, which are engaged in proprioception, body perception, and pain perception and empathy (36–38); the putamen, which subserves motor, learning and motivational functions (39–41); and frontopolar cortex, which inhibits the DMN and mediates among other intrinsic connectivity networks (42). Some areas have been implicated in regional pain syndromes, such as maxillofacial pain (e.g., angular and transverse temporal gyri) and may respond in a graded fashion as a function of pain intensity (e.g., frontopolar cortex) (43–45).

Previous attempts to characterize the connectivity of regions subserving pain perception and its sequelae have generally been limited to the well-defined CN V-TC and PAG-RVM pathways, LPS and MPS, DMN, and several of the miscellaneous or ancillary regions (5, 16, 26, 46–51). Adhering to the definition of the pain neuromatrix suggested by Melzack (9, 52), however, which includes *all* systems jointly activated by pain sensation and associated with perceptual-attentional, cognitive, affective, motivational, and nociceptive functions, little is known about its global topology. Specifically, it is unclear which connections have primacy (serve as network hubs) or how this topology changes in the presence of chronic pain syndromes. This exploratory study leverages resting-state functional MRI (rsfMRI) to begin to address these issues in a cohort of pain-free controls and individuals with painful TMD under basal and evoked-pain conditions, resulting in a preliminary description of the nociceptive network topology and its alteration in chronic pain conditions. TMD is one of several chronic overlapping pain conditions believed to be driven, at least in part, by central nervous system alterations. Fibromyalgia is considered by most to be a prototypical nociplastic pain condition, and higher “fibromyalgianess” scores have been found to be associated with more complex pain mechanisms and poorer outcomes in TMD and other chronic pain conditions (53–56). Therefore, in addition to the comparisons between pain and pain-free groups, we also assessed whether TMD patients degree of likely nociplastic pain (assessed with the fibromyalgia survey criteria) is correlated with the degree of central nervous system changes. The present study builds upon prior work utilizing rsfMRI and task-based fMRI which demonstrated changes in the CN V-TC, LPS and MPS, motor system, and DMN in individuals with TMD (see, for example, the recent review by 5).

## Methods

### Study population and testing

This study was approved by the University of Michigan Institutional Review Board and all subjects provided informed consent. 16 patients diagnosed with painful TMD (15 female, 1 male, aged 18–49 years) and 12 pain-free controls (11 female, 1 male, aged 19–57 years) participated. Participants with TMD were recruited from the Oral and Maxillofacial Surgery Center and Hospital Dentistry at the University of Michigan and controls were recruited through the University's online research match system. Two experienced physicians determined if participants with TMD met the diagnostic criteria and patients were subsequently included if they had nonspecific pain in the jaw/cheek area for a least 6 months. Pain-free control participants were excluded if they had a history of chronic pain lasting more than 6 months. All participants were excluded if they had physical impairments (e.g., amputation, blindness or deafness), severe psychiatric illnesses (e.g., major depression with suicidal ideation, schizophrenia, or substance abuse within the last 2 years), pregnancy, current tobacco use (>5 cigarettes/day, equivalent tobacco containing product), history of alcoholism, use of opioids including tramadol, sedatives including benzodiazepines or hypnotics, NMDA receptor antagonists (e.g., memantine), and eugeroics (e.g., modafinal) in the absence of appropriate washout periods, and the unstable use of the following medications: monoamine oxidase inhibitors (MAOIs), tricyclic antidepressants, selective serotonin reuptake inhibitors (SSRIs), serotonin norepinephrine reuptake inhibitors (SNRIs), antiepileptics, muscle relaxants. TMD and control groups did not differ with respect to age (p=0.399, Mann–Whitney *U* test). Participants were administered the Pennebaker Inventory of Limbic Languidness (PILL) (57), which yielded a hypervigilance score, as well as the 2010/2011 American College of Rheumatology Fibromyalgia Survey (ACRFS) (58, 59), which yielded widespread pain index (WPI), symptom severity (SS), and total ACRFS scores. Hypervigilance, WPI, and SS scores are subjective measures of patients' attention to aversive sensations, distribution of pain among 19 body areas, and general and domain-specific effects of pain symptoms, respectively (57, 59–61). Scores, as well as demographic and pain rating data, are summarized in Table 1.

**Table 1:** Demographic and clinical data.

	Control	TMD	MW U	*p*
Total N	12	16	Not applicable	* *
N female	11	15		
Age	36 (31)	32 (20)	72.500	0.399
Widespread pain index (WPI)	0 (1)	3 (3)	178.000	<0.001
Symptom severity (SS)	1.00 (2.00)	4.00 (5.00)	149.000	0.001
Hypervigilance (Pennebaker)	6.00 (8.00)	14.00 (13.00)	156.000	0.001
ACRFS total score	2 (3)	7 (9)	162.000	<0.001
Cuff pressure used	180 (60)	180 (40)	96.500	0.755
Pain rating	40 (23)	40 (40)	103.500	0.516

Normality was assessed by Kolmogorov–Smirnov tests, and non-normality indicated by italics. All results are reported as “median (intraquartile range)” and group differences assessed by Mann–Whitney *U* (*MW U*).

### MRI acquisition and preprocessing

Two fMRI datasets were acquired during the MRI session. Before the start of the scan, a 13.5 cm-wide velcro-adjusted pressure cuff, connected to a rapid cuff inflator (Hokanson E20 AG101, Hokanson Inc, Bellevue, WA, USA), was attached to the leg over the left gastrocnemius muscle, with the top of the cuff ∼4 cm distal to the bottom of the kneecap. The first rsfMRI scan was conducted with the cuff deflated (i.e., 0 mmHg). The second resting state scan was conducted with the cuff inflated to 200 mmHg; except, in cases where that pressure was intolerable, a lower pressure was used. Tolerability was determined by a 1-min inflation of the cuff outside of the scanner and again more briefly (5-s inflation) just before the start of the scan. If the pressure was intolerable, the pressure was adjusted in 20 mmHg increments until the participant found it tolerable. The cuff was inflated simultaneously with the start of the scan and deflated at the end. Perceived pain of the cuff inflation was obtained by having participants rate painfulness of the cuff on a scale from 0 to 100, where 0 means “no pain” and 100 means “the most intense pain imaginable.” These ratings were collected immediately following the cuff inflated and deflated runs.

Cuff-deflated and cuff-inflated (induced-pain) rsfMRI data were acquired as separate but consecutive scans (conditions) within a single imaging session on a 3.0 T GE Discovery MR750 (GE Medical Systems, Waukesha, Wisconsin) with a 32-channel Nova head coil. Echo-planar (EPI) rsfMRI data was acquired over a continuous 5-min period (resolution 2.386 mm × 2.386 mm × 2.500 mm, interleaved slices, TR = 1,200 ms, TE = 30 ms, FA = 70°, FOV = 210 mm, NEX = 1, multiband factor = 3). Two gradient-echo field maps (6.5 and 8.5 ms TE) and one T1-weighted 3D-spoiled gradient recalled echo (SPGR) structural scan (650 ms TR, 14.653 ms TE, 90° flip angle, echo train length (ETL) = 28) were also acquired for each subject. rsfMRI data was subjected to the default minimal preprocessing and denoising pipelines in the CONN Toolbox v19c (62, 63). These pipelines are described in detail in the Supplementary Material. All structural and denoised functional data, grey matter, white matter, and CSF masks, as well as regions of interest (see below), were manually inspected for quality assurance and to confirm registration validity. Selected quality assurance results are also presented in the Supplementary Material.

### Surface-based morphometry (SBM) and SBM regions of interest

Volumetric segmentation of the T1-weighted MPRAGE was performed with Freesurfer (v6.0: surfer.nmr.mgh.harvard.edu). The Freesurfer segmentation processing stream, which is described in detail elsewhere (64–67), consists of segmentation of grey/white matter and grey/cerebrospinal fluid tissue classes as well as the subcortical white matter and deep gray matter volumetric structures, and is not restricted to the voxel resolution of the original data, but is capable of detecting submillimeter differences between groups (64, 67–70). Freesurfer's “cross-sectional” workflow consists of automated brain extraction, segmentation, intensity normalization, and topological correction using intensity and surface continuity information from two channels represented by participants' T1 and surface continuity information from participants' T1-weighted images (71). Statistical evaluations of grey and white matter differences between the control and TMD groups were performed at the cluster level by multivariate general linear model (GLM) using Freesurfer's *Qdec* utility as well as via ROI-based GLM analyses in SPSS. A smoothing kernel of 10 mm full width at half maximum (FWHM) was applied to all segmentations prior to statistical analysis.

#### Cluster-based analyses

For cluster-based analyses, two sets of GLMs were conducted, the first as a group-wise analysis using a binary encoding of “group” as a fixed factor and “age” and total estimated intracranial volume (“eTIV”) as covariates, and the second as a regression-type analysis using ACRFS total scores, WPI, symptom severity, or Pennebaker scores as predictor variables and “age” and “eTIV” as covariates. In both cases, correction for multiple comparisons was performed by Monte Carlo simulations at $Z\geq 1.30\;∼\;\mathrm{family}-\mathrm{wise}\;\mathrm{error}\;(\mathrm{PFWE})\;P_{FWE}\leq 0.05$. Six suprathreshold clusters, including the left pre- and postcentral gyri (L PreCG, L PostCG), left supramarginal gyrus (L SMG), and left superior temporal sulcus and temporo-occipital and posterior middle temporal gyri (L toMTG, L pMiTG, L bSTS) were indicated as “of interest” by these cluster-based SBM analyses (see “Results” section); of these, L SMG was already included as an atlas-defined ROI. L PreCG, L PostCG, L toMTG, and L pMiTG were back-projected to subject space as volumetric regions of interest (ROIs) and leveraged in connectivity analyses, as described below, and included in Supplementary Table S1.

### *A priori* regions of interest

Additional ROIs for the present study were selected for their association with the trigemino-thalamocortical, or CN V-TC, pathway (5, 6), periaqueductal grey-rostral ventromedial medulla, or PAG-RVM, pathway (5, 72–75), lateral and/or medial, or LPS and MPS, pain systems (5, 7, 8, 76), the default-mode network, or DMN (17–22), or other components of the “pain neuromatrix” (5, 7, 9–11, 13–16, 77). These ROIs are listed in Supplementary Table S1. Although most ROIs were predefined in the CONN Toolbox as per the AAL and FSL Harvard-Oxford Maximum Likelihood Cortical and Subcortical atlases (78–81), several required definition from MNI coordinates reported in prior literature or were more precisely delineated from direct segmentation of participants' T1 volumes using Freesurfer 6.0 or SUIT v3.4 (82–86). Coordinate-defined ROIs included the periaqueductal grey (PAG), which was comprised of a medial, 6 mm-radius spherical ROI centered at MNI coordinate [0,−32,−12] per Coulombe, Lawrence et al. (87), and lateral and medial hypothalamus ROIs, comprised of 2 mm-radius spherical ROIs centered at [±6, −9, −10] and [±4, −2, −12], respectively, per Li et al. (88) Rostral and caudal anterior cingulate (rACC, cACC), brainstem structures [medulla, pons, midbrain, and superior cerebellar peduncle (SCP)], hippocampus (HC), amygdala (AMG), and entorhinal (EnRC) and anterior and posterior parahippocampal cortices (aPaHC, pPaHC) were delineated from T1 segmentations in Freesurfer and SUIT. In all cases, registration validity of stereotactically-defined ROIs was confirmed by visual inspection against individual structural data following back-projection to subject space. The total corpus from all sources was comprised of 86 ROIs.

### ROI-ROI connectivity matrix generation and inference testing

Four connectivity matrices were generated (control-deflated, control-inflated, TMD-deflated, and TMD-inflated) by computing the matrix Z of Fisher-transformed, bivariate correlation coefficients among the 86 ROIs (see Supplementary Table S1) using the CONN Toolbox. These coefficients were computed from the blood oxygenation level dependent (BOLD) BOLD timeseries within each pair of ROIs $R_{i}$ and $R_{j}$:

$$Z\left(i,j\right)=\tanh^{-1}R\left(i,j\right)$$

where

$$R\left(i,j\right)=\frac{\int R_{i}\left(t\right)R_{j}\left(t\right)dt}{\sqrt{\int R_{i}^{2}\left(t\right)dt\int R_{j}^{2}\left(t\right)dt}}$$

Assessments of functional connectivity as a function of group, condition, and Pennebaker and ACRFS measures were performed via GLM. For first-level GLM analyses, design matrices comprising *group* (control and TMD), *condition* (cuff-deflated and cuff-inflated), and *hypervigilance*, *WPI*, and *symptom severity* were defined while controlling for physiological and motion artefacts using the motion parameters generated during preprocessing. Second-level, within-subjects analyses on *condition* were conducted by leveraging individual ROI-ROI connectivity matrices within either the control (control inflated versus deflated) or TMD group (TMD inflated versus deflated) in paired-samples *t*-tests. Second-level, between-subjects analyses on *group* leveraged individual contrast images within either the deflated (TMD versus control while deflated) or inflated (TMD versus control while inflated) in independent-samples *t*-tests. Finally, second-level analyses against *hypervigilance*, *WPI*, and *symptom severity* scores were conducted as regression-type GLMs across all subjects and conditions. One analysis was performed for each of these scores by specifying *hypervigilance*, *WPI*, or *symptom severity* score as the predictor variable and ROI-ROI connectivity as the dependent variable. All regression models included an intercept term.

Inference testing for all GLM analyses was performed using network-based statistics (NBS), which treats ROI-ROI connectivity matrices as network graphs and subgraphs and extend the principles of cluster-based thresholding (89–91) to the graph model. The NBS approach controls for the familywise error rate *for each subgraph*, thereby increasing the contrast-to-noise ratio (92). These attributes are particularly attractive for the present study, wherein the correlation between an effect (group, condition, or Pennebaker or ACRFS scores) and topology of specific networks, rather than pairwise connection strengths between ROIs, is of interest, and where the contrast-to-noise ratio is of critical importance due to small sample size. Computation of the NBS proceeds by first applying an *a priori* “height” threshold (p<0.001, uncorrected) to the ROI-ROI matrix of *t*- or *F*-statistics to yield a suprathreshold network among all ROIs. Second, this network is decomposed, by breadth-first search, into component subgraphs which are each characterized by a “network mass,” defined as the sum of $t^{2}$- or *F*-statistics over all connections within the subgraph, and the mass of each subgraph compared with a null distribution estimated from multiple permutations of the initial data. Third, subgraph-level FWE-corrected *p*-values (the likelihood of finding a subgraph with the same or larger mass across all ROI-ROI connections) and false discovery rate (FDR) FDR-corrected *p*-values (the expected proportion of false discoveries among networks with the same or larger mass across all ROI-ROI connections) are computed.

### Adjacency matrix generation and graph-theoretic analyses

Group × condition adjacency matrices (control deflated, control inflated, TMD deflated, and TMD inflated) were generated from one-sample permutation tests against zero and corrected for multiple comparisons using NBS in the CONN Toolbox, as described previously (92–94). Suprathreshold and subthreshold connections were binary-encoded as 1 and 0 respectively, and a “core” adjacency matrix, consisting of connections which were suprathreshold across groups and conditions, generated by elementwise multiplication of the control-deflated, control-inflated, TMD-deflated, and TMD-inflated adjacency matrices. Nodal degree and betweenness centrality were also computed from the four group × condition adjacency matrices (but reported only for “core” connections in the “Results” section) using the Brain Connectivity Toolbox (95). These graph-theoretic metrics have been described in detail previously, and the reader is referred to those publications for a full discussion of the theoretical basis of the metrics (95–99). They are also described in detail in the Supplementary Material.

To assess differences in betweenness centrality and nodal degree among well-defined systems and pathways, ROIs were categorized as CN V-TC, PAG-RVM, LPS, MPS, or DMN, and a linear mixed-effects model conducted in Python *statsmodels* version 0.12.2 (100). Other ROIs (“pain neuromatrix” or “not otherwise specified”) were excluded from this analysis due to the ambiguity of their role in nociceptive processing. Separate linear mixed-effects models were conducted for the dependent variables *centrality* and *degree*. Each model was specified as a variance component model (101) using *group* (control or TMD) as the between-subjects factor and *condition* (deflated or inflated) and *ROI* “*family*” (CN V-TC, PAG-RVM, LPS, MPS, or DMN) as nested within-subjects factors, i.e.,

DV∼group+condition+family+group:condition:family

where a colon represents nested factors and *DV* the dependent variable (centrality or degree), and the variance component specified as

group=0+C(group),condition=0+C(condition),family=0+C(family),

where 0 represents a random intercept and *C* denotes the categorical nature of the variable. Contrasts for “ROI family” were specified as “family” versus “non-family,” e.g., “LPS ROIs” versus “non-LPS ROIs” (all ROIs not specified as having membership within the LPS). In this example, the mixed-effects model would be specified with *group* (control = 0; TMD = 1), *condition* (deflated = 0; inflated = 1), and LPS (true = 0; false = 1) as nested variables and betweenness centrality or nodal degree as continuous dependent variables.

Finally, resolution of communities among nodes in the four adjacency matrices and “core” adjacency matrix was estimated in the Brain Connectivity Toolbox using the Louvain community detection algorithm.

## Results

### ACRFS and Pennebaker scores

TMD participants demonstrated more widespread pain [*WPI*: control median ± intraquartile range (IQR), 0 ± 1, TMD 3 ± 3, p<0.001, Mann–Whitney *U* test], greater sensitivity to aversive sensations (*hypervigilance*: control median ± IQR, 6.00 ± 8.00, TMD 14.00 ± 13.00, p=0.001, Mann–Whitney *U*), and heightened effects of pain on fatigue, cognitive symptoms, and other domains (*symptom severity*: control median ± IQR, 1.00 ± 2.00, TMD 4.00 ± 5.00, p=0.001, Mann–Whitney *U*), relative to controls. ACRFS total scores also differed between control (median ± IQR, 2.00 ± 3.00) and TMD participants (7.00 ± 9.00, p<0.001, Mann–Whitney *U*). Cuff pressure was statistically equivalent in both groups (control median ± IQR, 180.00 ± 60.00 mmHg, TMD 180.00 ± 40.00 mmHg, p=0.755, Mann–Whitney *U*) as was perceived pain intensity (control median ± IQR, 40.00 ± 23.00, TMD 40.00 ± 40.00, p=0.516, Mann–Whitney *U*; see Table 1).

### Surface-based morphometry analysis

**Figure 1:**
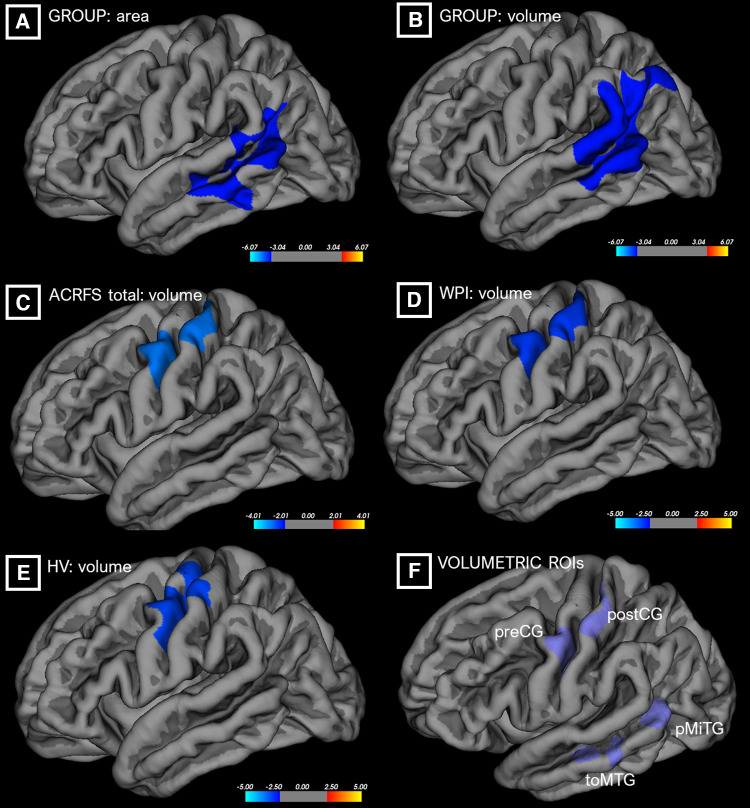
Differences in cortical surface area (“area,” **A**) or grey matter volume (“volume,” **B–E**) as a function of group or ACRFS scores, from cluster-based morphometric analyses. ROIs from these analyses (**F**) were incorporated into the connectivity workflows. ACRFS, American College of Rheumatology Fibromyalgia Survey; HV, Pennebaker Hypervigilance score; WPI, ACRFS Widespread Pain Index score.

The results of the SBM cluster-based analyses are shown in Figure 1. Control and TMD groups did not differ significantly with respect to total eTIV (F = 1.476, *p* = 0.237). A GLM on group, controlling for eTIV and corrected for FWE by Monte Carlo simulation, indicated that TMD subjects exhibited decreased left-hemisphere superior temporal surface area relative to controls. The center of mass of this cluster, at MNI [−54, −25, −6], was localized to the ventral-posterior aspect of the superior temporal sulcus. TMD subjects also exhibited reduced left superior temporal volume with a center of mass coordinate of [−64, −49, 15], which was localized to the supramarginal gyrus. Additional GLMs, conducted across all subjects as regressions, indicated that higher ACRFS WPI and ACRFS total scores were associated with decreased white matter volumes deep to the left parietal lobe, with centers of mass located at [−27, −19, 30] (corticospinal tract/superior corona radiata) for both effects, and that higher Pennebaker hypervigilance scores were associated with decreased grey matter volume across the left precentral and postcentral gyri, or the dorsal aspect of Brodmann area 6, with a center of mass located at [−39, −19, 67]. The analyses failed to resolve any effects of symptom severity on either grey or white matter volume, nor were any effects, among group, ACRFS total scores, Pennebaker scores, or age, associated with an increase or decrease in cortical thickness or area.

### ROI-ROI connectivity and graph theory analysis

#### Group×condition connectivity matrices

**Figure 2:**
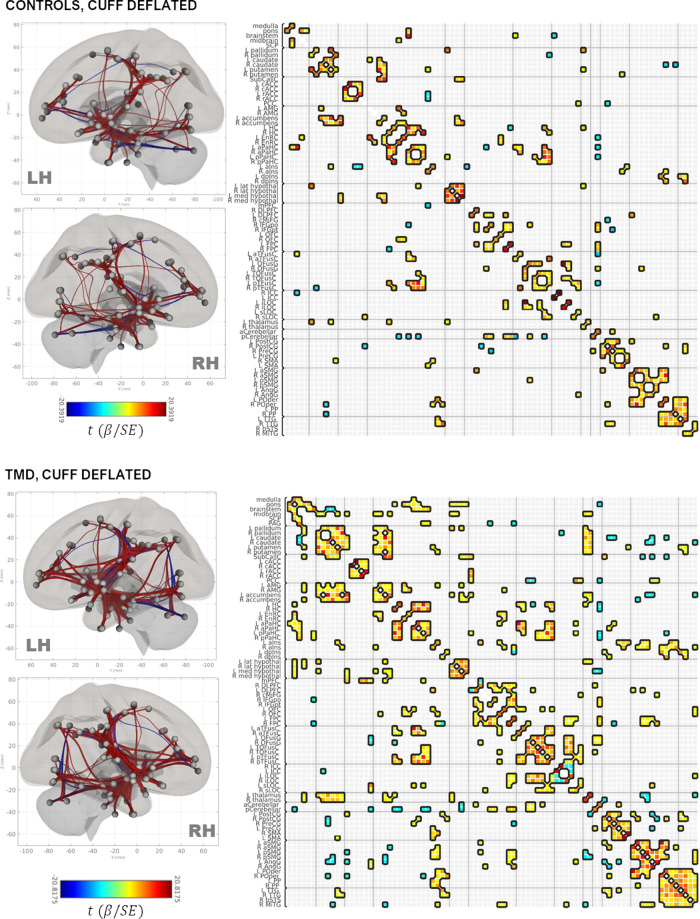
Rendering of functional connectivity networks and correlation matrices for connections surviving one-sample *t*-tests in control (**top panel**) and TMD participants (**bottom panel**) in the cuff-deflated condition. The color bar corresponds to the Wald *t*-statistic for each connection (the general linear model regression coefficient divided by its standard error). Negative, or blue, values represent negative or antiphase correlations between ROIs, whereas positive, or red values represent positive or in-phase correlations between ROIs. ROI abbreviations are defined in Supplementary Table S1.

**Figure 3:**
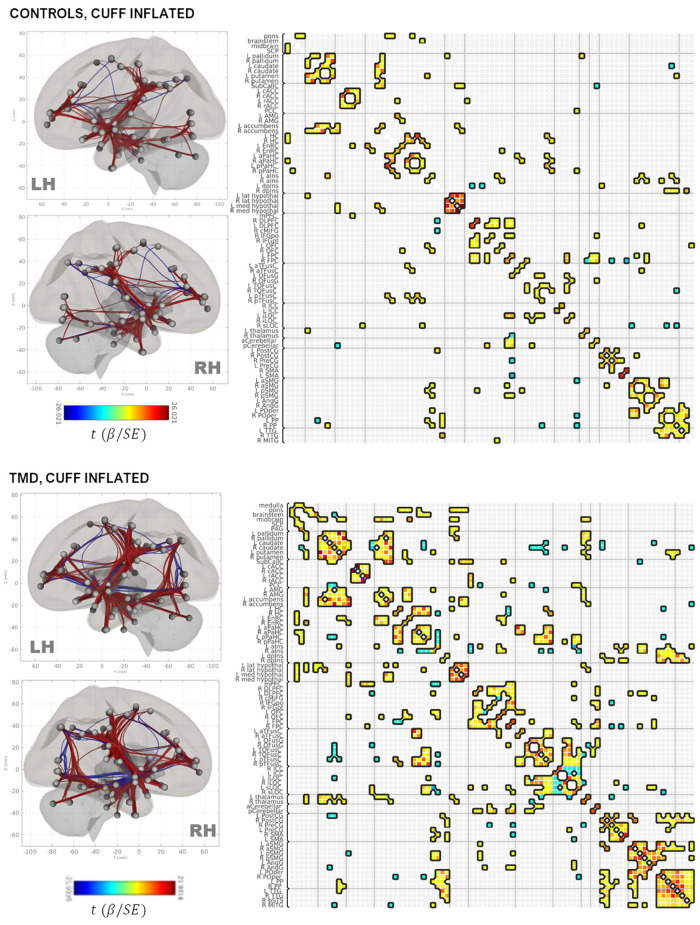
Rendering of functional connectivity networks and correlation matrices for connections surviving one-sample *t*-tests in control (**top panel**) and TMD participants (**bottom panel**) in the cuff-inflated condition. The color bar corresponds to the Wald *t*-statistic for each connection (the general linear model regression coefficient divided by its standard error). Negative, or blue, values represent negative or antiphase correlations between ROIs, whereas positive, or red values represent positive or in-phase correlations between ROIs. ROI abbreviations are defined in Supplementary Table S1.

Group adjacency matrices are presented in Figure 2 (deflated condition) and Figure 3 (inflated condition). Connections were considered “present” in a group/condition if they were suprathreshold following NBS, and “absent” otherwise. Across groups and conditions, adjacencies were noted within functionally and anatomically homologous ROIs. These ROIs were grouped into ten major classes: (1) brainstem and rhombencephalic structures (medulla, pons, brainstem, superior cerebellar peduncle); (2) basal ganglia; (3) cingulate structures (subcallosal cortex, posterior cingulate and rostral and caudal anterior cingulate); (4) medial temporal and associated structures (amygdala, accumbens, entorhinal and parahippocampal cortices, hippocampus, and anterior and dorsal posterior insula); (5) frontal lobe (dorsolateral prefrontal, caudal middle frontal, inferior frontal, orbitofrontal, and frontopolar cortices); (6) fusiform areas; (7) occipital lobe; (8) pre-, postcentral, and supplementary motor areas; (9) supramarginal and angular gyri, planum polare, and parietal operculum; and (10) temporal areas (transverse temporal and right middle temporal gyri and the bank of the superior temporal sulcus). Additionally, thalamic, hypothalamic, and cerebellar ROIs comprised minor classes.

**Figure 4:**
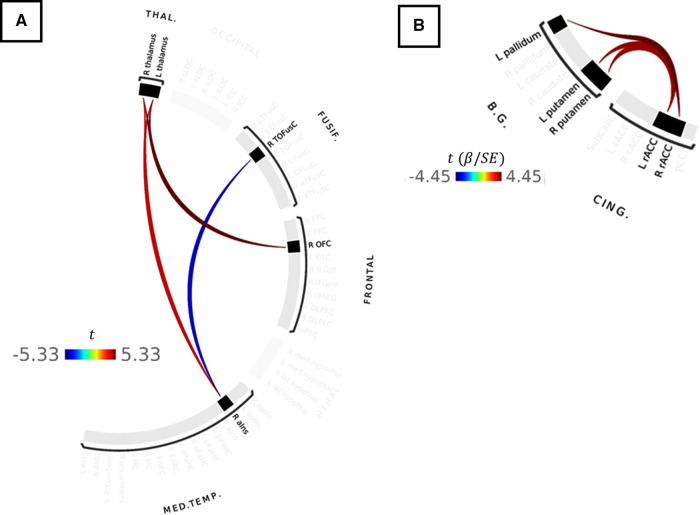
(**A**) Results of paired-samples *t*-test between cuff-inflated and cuff-deflated conditions for TMD participants. Red colors indicate stronger correlations between ROIs in the inflated condition, compared to deflated, whereas blue colors indicate weaker ROI-ROI correlations in the inflated condition compared to deflated. Color bar indicates the *t*-statistic. (**B**) Correlations between ROI-ROI connection strength and symptom severity in the inflated condition for all participants. Color bar indicates the Wald *t*-statistic for the GLM coefficient. No statistically-significant differences in connectivity strength were observed between TMD or control participants in the inflated or deflated conditions, or within TMD or control participants as a function of Pennebaker Hypervigilance, ACRFS Symptom Severity, or ACRFS WPI scores following correction for multiple comparisons using network-based statistics.

Connections between the basal ganglia and accumbens nuclei, basal ganglia and posterior temporal fusiform cortex, and posterior temporal fusiform cortex and superior and inferior lateral occipital cortices were present in the deflated condition in both groups. TMD participants exhibited more extensive connections overall, but particularly with respect to thalamic, medial temporal, rhombencephalic, frontal, temporal, and occipital ROIs, and more amygdalar connections with caudate, pallidum, entorhinal, and right anterior insula were present than in the control participants. In the inflated condition, control participants exhibited bilateral connections between pallidum, caudate, and right accumbens, left and right hippocampus and pons, inferior frontal and dorsolateral prefrontal, thalamus and frontopolar, and frontopolar and dorsolateral prefrontal ROIs, which were not present in the deflated condition, whereas connections between left anterior parahippocampal areas, the pons, and the brainstem, between the angular and supramarginal gyri, and between the right accumbens, left entorhinal, and right entorhinal ROIs, were absent. Conversely, TMD participants exhibited connections between fusiform cortex, caudate, and pallidum, left anterior insula and parahippocampal cortices, and between the amygdala, entorhinal, and left thalamus ROIs which were not present in the deflated condition, whereas connections between left anterior parahippocampal and rhombencephalic ROIs were absent, and more connections were present in TMD participants than control participants in the inflated condition. Connection *strength* did not differ significantly in a between-groups, within-condition analysis against either condition, or in a within-groups, between-condition analysis against the control participants. Within TMD participants, however, thalamic-right anterior insular and thalamic-right orbitofrontal connections were stronger (L thalamus-right aINS t=+4.47, $P_{unc}=0.004$, R thalamus-R aINS, t=+4.14, $P_{unc}=0.0008$), and right anterior insular-right temporo-occipital fusiform connections weaker (t=−4.41, $P_{unc}=0.0004$), in the inflated condition relative to the deflated condition (see Figure 4A and Table 2B; control participants were not included in Table 2B as there were no differences as a function of condition). These associations were significant at the connection level ($P_{unc}$ values listed above) and for the NBS “mass” of the pallidum-putamen-rostral anterior cingulate (graph $P_{FWE}$ = 0.0350) and orbitofrontal-thalamus-fusiform-anterior insula networks (graph $P_{FWE}$ = 0.0170), but not for their discrete subgraphs (subgraph $P_{FWE}$ = 0.1464–0.4919).

**Table 2:** Results of general linear model analyses against connectivity.

ROI1	ROI2	*t*	p(unc)	p(FWE)
**(A) Connectivity versus symptom severity, all participants**				
L pallidum	RH rACC	4.45	0.0002	0.4919
L pallidum	LH rACC	4.12	0.0004	0.4919
L putamen	RH rACC	4.02	0.0005	0.4919
R putamen	LH rACC	4.01	0.0006	0.4919
L putamen	LH rACC	3.86	0.0008	0.4919
R putamen	RH rACC	3.79	0.0009	0.4919
Network mass = 196.53, p(FDR) = 0.02095		0.0105	0.0350	
**(B) Connectivity in inflated > deflated condition, TMD participants**				
R OFC	R thalamus	5.33	0.0001	0.1464
R OFC	L thalamus	5.25	0.0001	0.1464
R aINS	L thalamus	4.47	0.0004	0.3195
R aINS	R TOFusC	−4.41	0.0004	0.3195
R aINS	R thalamus	4.14	0.0008	0.4025
Network mass = 224.98, p(FDR) = 0.0170		0.0188	0.0170	

(A) Functional connectivity as a function of symptom severity across all participants, conducted as an ordinary least-squares regression-type GLM, with a single intercept term, using symptom severity as the predictor variable; for this analysis, *t*-statistics represent regression coefficients divided by the coefficients' standard errors. (B) Comparison of functional connectivity within TMD participants in the cuff-deflated and cuff-inflated conditions, conducted as a *t*-test, inflated > deflated. Positive *t*-statistics represent connection strengths which were greater (stronger or more positive) in the inflated condition, and negative *t*-statistics represent connection strengths which were greater (stronger or more positive) in the deflated condition. Statistical significance and correction for multiple comparisons were conducted primarily at the network level using familywise error rate, and secondarily at the connection level using uncorrected *p*-values (92). Thus, p(FWE) refers to the familywise error rate of the subnetwork, p(unc) to specific connections within a subnetwork, and p(FDR), in the summary line, to the false discovery rate for the network comprised of the significant subnetworks and their connections. ROI abbreviations are as shown in Table 2.

#### Functional connectivity versus ACRFS and Pennebaker scores

GLM analyses of ACRFS and Pennebaker scores failed to resolve statistically-significant differences in functional connectivity as a function of hypervigilance, widespread pain index, symptom severity, or ACRFS total score following FWE correction with NBS. However, connection strength within one network motif, comprised of basal ganglia and cingulate ROIs, was observed to increase with symptom severity score when assessed across all participants. In particular, participants with higher symptom severity scores tended to exhibit stronger connections between rostral anterior cingulate and the pallidum (R rostral ACC [RH rACC]-L pallidum, $t=+4.45,\;\;P_{unc}=0.0002$, L rostral ACC [LH rACC]-L pallidum, $t=+4.12,\;\;P_{unc}=0.0004$) and putamen (RH rACC-L putamen, $t=+4.02,\;\;P_{unc}=0.0005$, LH rACC-R putamen, $t=+4.01,\;\;P_{unc}=0.0006$, LH rACC-L putamen, $t=+3.86,\;\;P_{unc}=0.0008$, RH rACC-R putamen, $t=+3.79,\;\;P_{unc}=0.0009$). These findings are presented as Figure 4B and Table 2A.

#### Group×condition adjacency matrices, “core” network, and graph theory analysis

**Figure 5:**
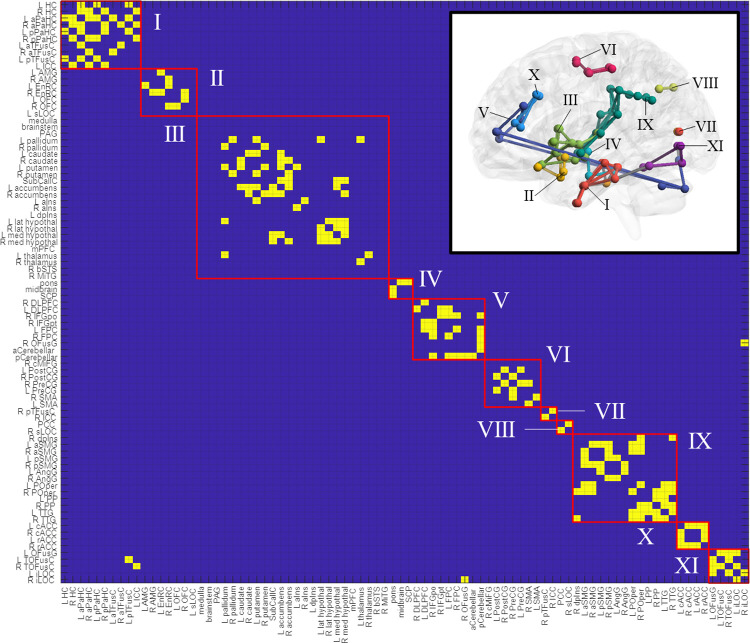
Nodal communities in the “core” network, as resolved with the louvain community detection algorithm. The core network is rendered in the figure inset. ROI abbreviations are defined in Supplementary Table S1.

Adjacency matrices for each group and condition were generated from suprathreshold connections following one-sample *t*-tests and NBS thresholding (Supplementary Figure S2). Connections present across groups and conditions were considered to constitute a “core” network, which was generated by a binary operation (elementwise multiplication) between the control-deflated, control-inflated, TMD-deflated, and TMD-inflated adjacency matrices (Supplementary Figure S2I) and rendered as Supplementary Figure S3 and the inset of Figure 5. Nodal communities within the core network, identified using the Louvain community detection algorithm, consisted of eleven classes: (I) hippocampus, anterior and posterior parahippocampal cortex, anterior and left posterior temporal fusiform cortex, and left intracalcarine cortex; (II) amygdala, entorhinal cortex, orbitofrontal cortex, and left superior lateral occipital cortex; (III) medulla, brainstem, periaqueductal grey, basal ganglia, subcallosal cortex, nucleus accumbens, anterior and left dorsal posterior insula, thalamus and hypothalamus, medial prefrontal cortex, the bank of the right superior temporal sulcus, and right middle temporal gyrus; (IV) pons, midbrain, superior cerebellar peduncle; (V) dorsolateral prefrontal cortex, right inferior frontal gyrus, frontopolar cortex, right occipital fusiform gyrus, and cerebellum; (VI) right caudal middle frontal gyrus, pre- and postcentral gyri, and supplementary motor cortex; (VII) right posterior temporal fusiform and ipsilateral intracalcarine cortex; (VIII) posterior cingulate and right superior lateral occipital cortex; (IX) right dorsal posterior insula, anterior and posterior supramarginal gyri, angular gyrus, parietal operculum, planum polare, and transverse temporal gyrus; (X) rostral and caudal anterior cingulate; and (XI) left occipital fusiform and left and right temporo-occipital fusiform cortex and the inferior lateral occipital cortex (Figure 5). Nodal communities were also detected for each group × condition adjacency matrix and are presented as Supplementary Figures S4, S5.

Finally, nodal degree and betweenness centrality were computed for each group × condition adjacency matrix and are presented as Supplementary Figure S6. Network “hubs,” identified by betweenness centrality rankings, included posterior cerebellum (betweenness centrality = 0.1774), right parietal operculum (0.1479), left anterior parahippocampal cortex (0.1372), and left putamen (0.1262) for controls under the deflated condition; left anterior insula (0.2013), right frontopolar cortex (0.1591), left entorhinal cortex (0.1360), left putamen (0.1349), right planum polare (0.1332), and left posterior supramarginal gyrus (0.1286) for controls under the inflated condition; left posterior parahippocampal cortex (0.0732), right frontopolar cortex (0.0721), right parietal operculum (0.0587), posterior cerebellum (0.0486), and left nucleus accumbens (0.0418) for TMD participants under the deflated condition; and right anterior insula (0.0871), right caudate (0.0776), right frontopolar cortex (0.0615), left posterior parahippocampal cortex (0.0557), and right parietal operculum (0.0507) for TMD participants under the inflated condition. These results suggest that the importance or “hub-ness” of the pons, right anterior insula, left parahippocampal cortex, left angular gyrus, and orbitofrontal cortex, among others, is amplified in TMD participants across conditions, despite lower centralities within the TMD group overall (Supplementary Figure S7).

**Figure 6:**
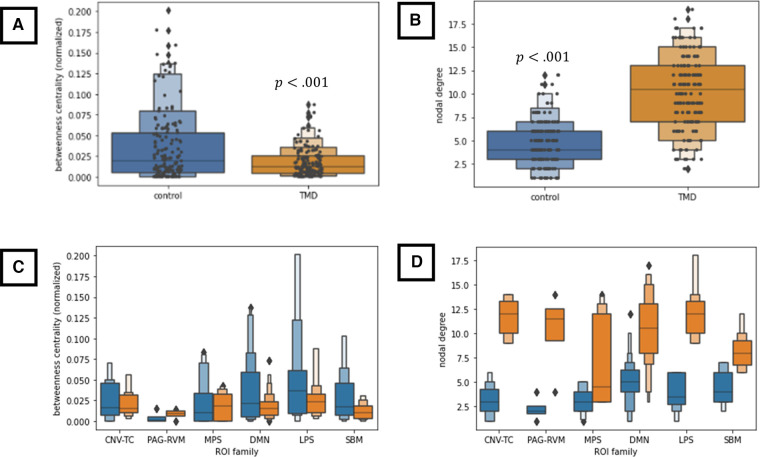
(**A,B**) Group-wise differences in node betweenness centrality (**A**) and nodal degree (**B**) across all nodes and both conditions. (**C,D**) Group-wise differences in betweenness centrality (**C**) and nodal degree (**D**) as a function of ROI family across both conditions. All results in this figure were computed using a linear mixed-effects model (see text), which is robust to violations of data normality. CNV-TC, trigemino-thalamocortical pathway; DMN, default-mode network; LPS, lateral pain system; MPS, medial pain system; PAG-RVM, periaqueductal grey-rostral ventral medulla pathway; SBM, derived from surface-based morphometry analyses. ROI membership in the various pain systems is defined in Supplementary Table S1. ROIs not assigned to the above pain systems (“not otherwise specified” or “NOS” in Supplementary Table S1) were not included in these analyses.

Differences in either degree or centrality were separately assessed for each “ROI family” (CN V-TC, PAG-RVM, LPS, MPS, or DMN) by linear mixed-effects models. Across ROI families and conditions, TMD participants exhibited lower betweenness centralities [mean ± standard deviation in the deflated condition 0.0173 ± 0.0166 versus 0.0346 ± 0.0410 in controls; inflated, 0.0175 ± 0.018 versus 0.0345 ± 0.042 in controls; F(1,306)=22.558, mean squared error (*MSE*) =0.023,t(306)=−4.75,p<0.001, $R_{adjusted}^{2}=0.066,\;\;\eta_{\;partial}^{2}=0.069\;$, Cohen's d=0.541] and higher nodal degree [deflated, 9.674 ± 3.981 versus 4.558 ± 2.655 in controls; inflated, 10.116 ± 4.143 versus 4.047 ± 2.415 in controls; F(1,306)=215.276,MSE=10.794,t(306)=14.672, $p<0.001,R_{adjusted}^{2}=0.413,\;\;\eta_{\;partial}^{2}=0.413$, Cohen's d=1.672] relative to control participants (Figure 6). Neither degree nor centrality differed within groups as a function of *condition* (deflated versus inflated) when taken across ROIs [centrality F(2,308)=0.0010, effect of *condition* within *group* at *inflated*: β=−0.017,z=−0.267, standard error (SE) 0.064, p=0.789; degree F(2,308)=10.816, effect of *condition* within *group* at *inflated*: β=5.935,z=0.899,SE=6.599,p=0.368], and consequently, additional nested effects and interactions were not explored. These findings therefore indicate strong effects of *group* on both node centrality and degree but failed to discern any meaningful differences within groups with respect to nodal centrality or degree in the cuff-deflated or cuff-inflated conditions.

## Discussion

This exploratory study represents an investigation of the network topology of systems and pathways subserving pain sensation, perception, discrimination, anticipation, learning and motivational functions, and affective function in control and TMD participants at rest and during evoked-pain conditions, with a principal objective of documenting the overall network structure as a function of group and condition, including identification of “hub” nodes and topological changes during induced pain in each group. An attempt was made to ensure that the major nociceptive pathways, including the CN V-TC, pPAG-RVM, LPS and MPS, and DMN, as well as components of the “pain neuromatrix,” such as hippocampus, fusiform cortex, striatum, frontopolar cortex, cerebellum, and ventral pallidum, were well-represented.

In the current cohort, TMD participants differed substantively from controls with respect to overall pain (ACRFS total score or “fibromyalgianess”) widespread pain index, sensitivity to aversive sensations (hypervigilance), and influence of pain on fatigue, cognition, and other quality-of-life aspects (symptom severity). By design, cuff pressure was equilibrated across individual participants, such that perceived pain intensity during the evoked-pain (cuff-inflated) condition was comparable between the two groups (Table 1).

### Morphometric findings

Surface- and volume-based assessments of grey and white matter structure were conducted primarily to generate additional ROIs which differed as a function of group or ACRFS and Pennebaker scores. Whole-brain/cluster-based and ROI-based morphometric analyses were conducted. These analyses evinced decreased left superior temporal surface area and volume (largely localized to the supramarginal gyrus), decreased surface areas in the right caudal middle frontal, right supramarginal, and right bank of the superior temporal sulcus, and increased right inferior frontal and right precuneus surface areas in TMD participants, as well as negative correlations between ACRFS total and widespread pain scores and corticospinal/corona radiata white matter volume, and a negative correlation between hypervigilance scores and grey matter volume in the pre- and postcentral gyri, across all subjects (Figure 1).

The significance and direction of structural findings differs across studies depending on covariates and other factors, and in general, such findings are inconsistent across studies, possibly due to individual differences in pain duration and history (5). Several studies have reported significant volumetric differences between TMD and controls [e.g., (102), and as reviewed in (47)]; for example, chronic pain is associated with grey matter alterations in thalamus and primary somatosensory cortex (47), and maxillofacial pain tolerance, in particular, has been associated with decreased grey matter volume in the right inferior frontal, right anterior insular, right putamen, left ventroposterior and right ventrolateral thalamus, and right globus pallidus, and increased grey matter volume in the ventral pons and middle cerebellar peduncle (5, 25, 47, 103). Additionally, the current results replicate several previous findings, including a reduction in superior (24) and middle temporal (104), medial prefrontal (105), and inferior frontal (24) grey matter volume. The reduction in inferior frontal surface area, as well as the negative associations currently observed between ACRFS total and WPI scores and corticospinal tract volume, and between hypervigilance scores and pre- and postcentral grey matter volume, may also comport with previously published observations that orbitofrontal grey matter thickness and primary motor cortical thickness are negatively associated with pain unpleasantness and duration, respectively (25, 106). Higher ACRFS scores were recently shown to be associated with higher levels of clinical pain, functional limitation, and disability and also with reduced responsiveness to arthroscopy of the temporomandibular joint in TMD patients (55, 56). These central findings could help explain why the clinical pain picture is often more complex in patients with higher fibromyalgianess. Interpretation of these findings is complicated by the deficiency in theoretical and clinical understanding of the causal (or acausal) relationship between pain and neural microanatomy. As noted by Younger, it is possible that these structural differences represent pre-existing vulnerability to pain or its chronic effects, or, alternatively, are secondary, adaptive responses to chronic pain; however, neither surface-based nor volume-based morphometry facilitates evaluation of critical aspects of neurite structure or glial density which would assist in such interpretation (25). Moreover, diffusion-based analyses and subcortical morphometry (including cerebellar morphometry) were omitted from the present study, thereby precluding examination of group-wise differences in these metrics in the present context. This omission should be addressed in future studies.

### Association between connectivity strength and pain scores

Although TMD and control participants in the present study differed substantively with respect to symptom severity, hypervigilance, WPI, and ACRFS total scores (Table 1), only symptom severity was associated with differences in the strength of functional connectivity when taken across all participants. *Symptom severity* is defined in the ACRFS as the impact of pain on fatigue, cognitive symptoms, the restorative nature of sleep, and other somatic symptoms over the previous 7 days and, in the current study, was positively associated with connectivity strength between the right rACC and contralateral pallidum as well as between the left rACC and left and right putamen (Figure 4B). In addition to their conventional role in the regulation of voluntary movement (39, 107, 108), both the putamen and the pallidum (particularly the ventral pallidum, a component of the dopaminergic reward system) subserve learning and motivational functions (33, 34, 40, 41). These functions may be effected, in part, *via* putamenal connections with association, sensorimotor, and limbic cortices, including the insula (39). The rACC is considered part of the MPS (5, 7, 8, 76) and a second-order perceptual component of the nociceptive-processing system (9), and is believed to subserve the affective and cognitive aspects of pain. The association between symptom severity and rACC-putamenal-pallidal connectivity in this study may therefore reflect the affective and motivational aspects of pain. Depression, which is a component of symptom severity, was recently found to be associated with connectivity between the prefrontal cortex, the insula, and other cortical regions in patients with chronic low back pain (109, 110).

In contrast to symptom severity, the ACRFS WPI and Pennebaker hypervigilance are related to the distribution of pain over the body, and the tendency to prioritize pain signals over other somatic or environmental cues (111, 112), respectively. Although these perceptual and attentional correlates should arguably be reflected in inter-regional connectivity, no effects of the WPI or hypervigilance measures on connectivity strength were detected, despite substantive differences in WPI and hypervigilance between groups. Kutch et al. (2017) found the WPI to be positively correlated with functional connectivity between the salience network and the somatosensory and motor cortex in patients with chronic pelvic pain (113). The lack of significant findings in the present study may be due to the small sample size of, or exclusion of key regions of interest from, the current study. Alternatively, increased hypervigilance and WPI may partially explain the groupwise differences in the resting (deflated) and evoked-pain (inflated) condition adjacency matrices (described below); however, this possibility was not explored.

Finally, it must be noted that symptom severity, WPI, hypervigilance and ACRFS total score reflect participant phenotypes and correspond to general aspects of pain severity and quality-of-life, rather than severity or perceived pain intensity during the MRI sessions. As cuff pressure and pain rating were equilibrated across participants in both the control and TMD groups, it is unlikely that differences in the strength of functional connectivity reflect differences in perceived pain intensity during the evoked-pain condition.

### Differences in connectivity strength as a function of group and condition

ROI-ROI connectivity strength was not observed to differ in the current cohort either between the control or TMD groups (across conditions) or between conditions in the control group. However, TMD participants demonstrated stronger connectivities between the left and right thalamus, right anterior insula, and right orbitofrontal cortex, and weaker connectivities between right anterior insula and the right temporo-occipital fusiform cortex, in the inflated condition compared to the deflated condition (Figure 4A). The anterior insula is a component of the MPS which functions in pain anticipation and salience, and may serve to modulate aversive, pain-related sensations *via* anticipatory downregulation of nociceptive regions (5, 7, 76, 114–117), and, in the present study, was correlated with both right and left thalamus ROIs but anticorrelated with the right fusiform cortex in TMD participants during the evoked condition. The fusiform cortex is known to function in body and face recognition and differentiation (30), functions which may be of particular interest in maxillofacial pain syndromes such as TMD. More generally, however, it has been shown to subserve pain anticipation and perception (31) and contributes to the affective and motivational aspects of pain. Like the striatum, accumbens, and orbitofrontal and prefrontal cortices, the fusiform cortex is part of the dopaminergic reward system (27–29) and is activated by stimulation of Aδ fibers (32) and during postoperative pain (118). Finally, the fourth component of this thalamic-anterior insular-fusiform-orbitofrontal network, the orbitofrontal cortex (OFC), also subserves pain inhibition and, with the accumbens nucleus, ventral striatum, dorsolateral prefrontal cortex, and thalamus (among others), is associated with motivational functions *via* the dopaminergic “reward circuit” (27–29, 119). The association of the components of this system with pain anticipation and modulation may thus indicate top-down effects on face-specific representations in the fusiform gyrus in anticipation of the evoked pain condition. However, no causal analyses were performed in this exploratory study, and such analyses could serve as a subject for future research.

### Graph-theoretic analyses

In the current study, group × condition adjacency matrices were based on one-sample tests to determine whether a given ROI-ROI connection was “present” (or suprathreshold) or “absent” in a given group and condition. The assessments of nodal degree and centrality, which are derived from these adjacency matrices, therefore differ from the correlation-based analyses of ROI-ROI connectivity strength described previously, not only with respect to approach but also to interpretation, despite the application of NBS thresholding to both one-sample and two-sample tests. An example is presented as Supplementary Figure S8, in which the normalized connectivity values of the L thalamus:R OFC connection, which differed in strength between conditions within TMD participants, are compared to those of the L PO (parietal operculum):L dpIns (dorsal-posterior insula) connection, which was “present” in the TMD-inflated adjacency matrix, but “absent” in the controls-inflated adjacency matrix, based on one-sample tests. In the paired-sample, L thalamus:R OFC case, TMD-deflated connectivities were negatively skewed, with a greater number of negatively-valued connections, whereas TMD-inflated connectivities were positively skewed, with a number of positively-valued, moderately negatively-valued, and highly negatively-valued connections, despite the Fisher normalization performed during data preprocessing. Consequently, the mean connectivity values in both conditions diverged from each other and from zero, yielding a significant result in the paired-samples test (see Supplementary Figure S8A and Figure 4A). Conversely, the L PO:L dpIns connectivity values in the inflated condition were slightly positively skewed for both control and TMD participants, indicating the presence of a slightly greater number of positively-valued connections in both groups, but only TMD participants' connectivities diverged (as a group) so substantively as to yield a suprathreshold one-sample test result (see Supplementary Figure S8B and Figure 3). One interpretation of this result is that, taken as a group, there was insufficient evidence to conclude that the correlation between the left parietal operculum and ipsilateral dorsal posterior insula ROIs was greater than the mean correlation among all ROIs (z=0) in the control participants during the inflated condition, whereas the statistical evidence did indicate a divergence from the mean ROI-ROI correlation in TMD participants. Conceptually, then, pairwise tests, which account for differences in two connectivity distributions, are useful for determining differences in ROI-ROI connectivity strengths between groups and conditions, whereas adjacency matrices, and the graph-theoretic metrics derived from them, are useful for describing and visualizing the network topology within each group and condition. Adjacency matrices are, however, subject to small variations in the distribution of connectivities due to the presence of a greater number of positive or negative ROI-ROI correlations in a group and condition, meaning that sample size is a strong determinant of the stability of the adjacency matrices; consequently, these results will need to be replicated with much larger samples than those used in the present, exploratory study.

For the current cohort of participants, network topology was generally conserved across groups and, to some degree, across conditions. Visual inspection of the adjacency matrices and renderings of this network in the deflated (Figure 2) and inflated conditions (Figure 3), as well as visual plots of the nodal community structure derived for connections which were present across groups and conditions (denoted in this paper as the “core” network: see Figure 5 and Supplementary Figure S3) provide an overall sense of its structure at rest and in the presence of evoked pain. Eight major and three minor functional subdivisions across subcortical areas and the prefrontal, temporal, cingulate, occipital, and parietal lobes, comprised the core network. The largest of these subdivisions was comprised of striatal, subcallosal (subgenual) cingulate, accumbens nuclei, bilateral anterior and left dorsal posterior insula, thalamus, and hypothalamus. Other major subdivisions included temporal and medial temporal structures and calcarine cortex; amygdala, orbitofrontal, and entorhinal cortex; the pons, midbrain, and the superior cerebellar peduncle; frontal/prefrontal and fusiform cortex and the cerebellum; middle frontal and somatomotor cortex; right dorsal posterior insula and parietal structures; rostral and caudal anterior cingulate; and temporo-occipital fusiform and inferior lateral occipital cortex. Notably, however, the density of connections in TMD participants across both conditions contributed to variations in community structure compared with controls (Supplementary Figures S4, S5).

Indeed, the connection density of TMD participants, across both the cuff-delated and cuff-inflated, or evoked pain, conditions is apparent throughout the current findings, including the number of connections “present” in the group × condition adjacency matrices (Figures 2, 3) and the marked decrease in nodal betweenness centrality (Figure 6 and Supplementary Figures S6, S7) and increase in nodal degree (Figure 6 and Supplementary Figure S6), in addition to the integration of nodes into functional communities. TMD nodal centralities and nodal degree were approximately 50% and 230%, respectively, of their observed values in control participants, rendering discrimination of “hub” nodes more difficult in the TMD group. For example, whereas control participants exhibited marked differences in node centrality between the two conditions, such that the primacy of the posterior cerebellum, right parietal operculum, and right nucleus accumbens were apparent in the cuff-deflated condition, and that of the left anterior insula, right prefrontal cortex, left orbitofrontal cortex, and other nodes in the cuff-inflated condition, fewer differences in node primacy were evident in TMD participants. Whereas the centrality of right anterior insula, right caudate, and right planum polare were markedly increased in TMD participants in the presence of evoked pain, for example, differences between conditions with respect to the centrality of the right frontopolar cortex, parietal operculum, and left entorhinal cortex were less substantive in this group (see Supplementary Figure S7; note the difference in *y*-axis scale). This loss of “hub-ness” is called “hub disruption” and has been documented in chronic pain syndromes such as migraine (120), back pain (121–124) and in rodent models of neuropathic pain (125, 126). Additionally, increased connectivity with respect to hubs such as the planum polare, occipital cortex, frontopolar cortex, and supramarginal gyri in episodic migraine (51, 127), and among default-mode components (medial prefrontal cortex, thalamus, posterior cingulate) (26) across various pain syndromes. Kaplan et al. (2019) identified the anterior insulae as being important hubs for processing in fibromyalgia patients, and they also discovered that hub membership of some pain-processing regions varied based on clinical pain intensity. The regional direction and specificity of hub disruption differs, however, depending on the nature of the pain syndrome and whether the data being analyzed is structural (128, 129) or functional, and it is as yet unclear whether such disruption is a contributor to, or a consequence of, chronic pain syndromes, and its interpretation may be region-specific. For example, Yin and colleagues have proposed that alterations in primary and supplementary motor cortices may be a function of “maladaptive neuroplasticity” (5), whereas other instances, such as aberrant connectivity between the prefrontal cortex, pregenual anterior cingulate, and amygdala [reviewed in (5)] and connectivity among default-mode components, which has been associated with pain rumination (26), are at least partially due to alterations in the interaction between nociceptive and attentional systems. Moreover, many of these studies also describe *decreases* in functional connectivity, such as decreased frontopolar activation (50) or reduced medial prefrontal-PAG connectivity (48), which were not reproduced in the present paper.

It should be noted that there are numerous methodological approaches to assessing functional connectivity, and such approaches may be more appropriate for some purposes than the NBS analysis of bivariate correlations used in the present paper. For example, recent research by He et al. (130) and Zhang et al. (49) have investigated the spectral power of low-frequency BOLD signals (fractional amplitude of low-frequency fluctuations, fALFF) and synchrony of spontaneous signals within brain areas (regional homogeneity, ReHo), respectively, to demonstrate improvements in local or regional function with gnathological treatment. These improvements were also correlated with subjective pain relief. Notably, these approaches are appropriate for evaluation of local brain activity, rather than distributed network activity, and should be viewed as complementary approaches with resting state.

There are several limitations to the current study which render it exploratory rather than conclusive. The most obvious of these is the small sample size, which is not only subject to sampling variability and affects statistical power, but also excludes the possibility of more interesting and powerful approaches to the characterization of participant phenotypes and network structure, such as clustering and graph similarity, and limits the present analyses to groupwise comparisons. Additionally, the FWER and FDR corrections for multiple comparisons in this small sample may be overly conservative and select for the largest effect sizes, limiting replicability (131). We attempted to address some of these concerns through the NBS approach, which focuses hypothesis tests on network topology over connection strength and enhances the contrast-to-noise ratio in smaller samples; however, the sample size also admits the possibility of model overspecification, particularly in the linear mixed effects modeling. Also, TMD duration was not included as a covariate due to the difficulty in accurately establishing the duration from self-report as well as the lack of a prior hypothesis regarding how this duration would affect the results. Nevertheless, this is an important consideration which will need to be addressed in a larger cohort. With most neuroimaging techniques in cross sectional studies, it is difficult to ascertain causation concerning relationships between symptoms and brain attributes. There is some evidence that perceived pain levels while at rest in the scanner are associated with functional connectivity (132, 133), meaning that comparisons made between individuals experiencing chronic pain while in the scanner and those experiencing no pain could be driving some group differences in resting state connectivity. Inclusion of a task (like inflation of the cuff) helps to determine causal changes in connectivity, but it does not solve the between-group comparison issues. Finally, it should be noted that network topology cannot be reliably ascertained using resting-state connectivity alone, but should be premised upon a combination of modalities, including structural analyses. Despite these limitations, however, it is our expectation that our thorough documentation of source ROIs, which were carefully selected to ensure adequate representation of major nociceptive systems as well as components of the ill-defined “pain neuromatrix,” will help facilitate future studies, including those which serve to replicate or refute our present findings.

## Conclusion

This pilot study represents a first attempt to leverage graph-theoretic analyses to evaluate the topology of the various nociceptive networks and systems, including those subserving pain sensation, perception, and discrimination, internal state and body perception, affective/motivational, and anticipatory functions, as a unified whole. Its results, which must be interpreted in the context of the small sample size, suggest that first-order nociceptive and second-order cognitive, affectual, and attentional systems comprise a distributed network located primarily in prefrontal, temporal, parietal, and cerebellar areas. Furthermore, the findings suggest that, although the *substrates* of this network do not generally differ between control and TMD participants in resting or evoked-pain conditions, the TMD syndrome may be associated with an overall increase in functional connectivity even at rest. Future studies will be required to replicate these results, incorporate structural connectivity, and establish causal associations among the network nodes delineated.

## Data Availability

The raw data supporting the conclusions of this article will be made available by the authors, without undue reservation.

## References

[1] Harper D Schrepf A Clauw D. Pain mechanisms and centralized pain in temporomandibular disorders. J Dent Res. (2016) 95(10):1102–8. 10.1177/0022034516657070 27422858 PMC5004242

[2] Zhang Z Gewandter JS Geha P. Brain imaging biomarkers for chronic pain. Front Neurol. (2021) 12:734821. p. 1–15. 10.3389/fneur.2021.734821 PMC8763372 35046881

[3] Lewis GN Rice DA McNair PJ. Conditioned pain modulation in populations with chronic pain: a systematic review and meta-analysis. J Pain. (2012) 13(10):936–44. 10.1016/j.jpain.2012.07.005 22981090

[4] Weaver KR Griffioen MA Klinedinst NJ Galik E Duarte AC Colloca L Quantitative sensory testing across chronic pain conditions and use in special populations. Front Pain Res. (2021) 2:779068. p. 1–9. 10.3389/fpain.2021.779068 PMC8915716 35295425

[5] Yin Y He S Xu J You W Li Q Long J The neuro-pathophysiology of temporomandibular disorders-related pain: a systematic review of structural and functional MRI studies. J Headache Pain. (2020) 21(1):78. 10.1186/s10194-020-01131-4 32560622 PMC7304152

[6] Shankland WE. The trigeminal nerve. Part II: the ophthalmic division. Cranio. (2001) 19(1):8–12. 10.1080/08869634.2001.11746145 11842844

[7] Tracey I. Nociceptive processing in the human brain. Curr Opin Neurobiol. (2005) 15(4):478–87. 10.1016/j.conb.2005.06.010 16019203

[8] Albe-Fessar D Berkley K Kruger L Ralston Iii H Willis W Jr. Diencephalic mechanisms of pain sensation. Brain Res Rev. (1985) 9(3):217–96. 10.1016/0165-0173(85)90013-X 3896408

[9] Garcia-Larrea L Peyron R. Pain matrices and neuropathic pain matrices: a review. Pain. (2013) 154(Suppl 1):S29–43. 10.1016/j.pain.2013.09.001 24021862

[10] Moseley GL. A pain neuromatrix approach to patients with chronic pain. Man Ther. (2003) 8(3):130–40. 10.1016/S1356-689X(03)00051-1 12909433

[11] Derbyshire SW. Exploring the pain “neuromatrix”. Curr Rev Pain. (2000) 4(6):467–77. 10.1007/s11916-000-0071-x 11060593

[12] Ianetti G. Electrocortical response to nociceptive stimulation in humans. Pain. (2010) Suppl 6:63–72.

[13] Melzack R. From the gate to the neuromatrix. Pain. (1999 Suppl 6):S121–S6. 10.1016/S0304-3959(99)00145-1 10491980

[14] Roy M Wager TD. Neuromatrix theory of pain. In: Jennifer C, editor. The Routledge handbook of philosophy of pain. London: Routledge (2017). p. 87–97.

[15] Hadjipavlou G Dunckley P Behrens TE Tracey I. Determining anatomical connectivities between cortical and brainstem pain processing regions in humans: a diffusion tensor imaging study in healthy controls. Pain. (2006) 123(1-2):169–78. 10.1016/j.pain.2006.02.027 16616418

[16] Festa F Rotelli C Scarano A Navarra R Caulo M Macri M. Functional magnetic resonance connectivity in patients with temporomadibular joint disorders. Front Neurol. (2021) 12:629211. 10.3389/fneur.2021.629211 33912123 PMC8072218

[17] Spreng RN Stevens WD Chamberlain JP Gilmore AW Schacter DL. Default network activity, coupled with the frontoparietal control network, supports goal-directed cognition. Neuroimage. (2010) 53(1):303–17. 10.1016/j.neuroimage.2010.06.016 20600998 PMC2914129

[18] Buckner RL Andrews-Hanna JR Schacter DL. The brain's default network: anatomy, function, and relevance to disease. Ann N Y Acad Sci. (2008) 1124:1–38. 10.1196/annals.1440.011 18400922

[19] Gusnard DA Raichle ME Raichle ME. Searching for a baseline: functional imaging and the resting human brain. Nat Rev Neurosci. (2001) 2(10):685–94. 10.1038/35094500 11584306

[20] Laird AR Eickhoff SB Li K Robin DA Glahn DC Fox PT. Investigating the functional heterogeneity of the default mode network using coordinate-based meta-analytic modeling. J Neurosci. (2009) 29(46):14496–505. 10.1523/JNEUROSCI.4004-09.2009 19923283 PMC2820256

[21] Shulman GL Fiez JA Corbetta M Buckner RL Miezin FM Raichle ME. Common blood flow changes across visual tasks: II. Decreases in cerebral cortex. J Cogn Neurosci. (1997) 9:648–63. 10.1162/jocn.1997.9.5.648 23965122

[22] Raichle ME MacLeod AM Snyder AZ Powers WJ Gusnard DA Shulman GL. A default mode of brain function. Proc Natl Acad Sci U S A. (2001) 98(2):676–82. 10.1073/pnas.98.2.676 11209064 PMC14647

[23] Lee J Andronesi OC Torrado-Carvajal A Ratai EM Loggia ML Weerasekera A 3D magnetic resonance spectroscopic imaging reveals links between brain metabolites and multidimensional pain features in fibromyalgia. Eur J Pain. (2021) 25(9):2050–64. 10.1002/ejp.1820 34102707 PMC9176690

[24] Gerstner GE Gracely RH Deebajah A Ichesco E Quintero A Clauw DJ Posterior insular molecular changes in myofascial pain. J Dent Res. (2012) 91(5):485–90. 10.1177/0022034512443366 22451533 PMC3327733

[25] Younger JW Shen YF Goddard G Mackey SC. Chronic myofascial temporomandibular pain is associated with neural abnormalities in the trigeminal and limbic systems. Pain. (2010) 149(2):222–8. 10.1016/j.pain.2010.01.006 20236763 PMC2860657

[26] Kucyi A Moayedi M Weissman-Fogel I Goldberg MB Freeman BV Tenenbaum HC Enhanced medial prefrontal-default mode network functional connectivity in chronic pain and its association with pain rumination. J Neurosci. (2014) 34(11):3969–75. 10.1523/JNEUROSCI.5055-13.2014 24623774 PMC6705280

[27] O'Doherty JP. Reward representations and reward-related learning in the human brain: insights from neuroimaging. Curr Opin Neurobiol. (2004) 14(6):769–76. 10.1016/j.conb.2004.10.016 15582382

[28] Haber SN Knutson B. The reward circuit: linking primate anatomy and human imaging. Neuropsychopharmacology. (2010) 35(1):4–26. 10.1038/npp.2009.129 19812543 PMC3055449

[29] Schultz W. Multiple reward signals in the brain. Nat Rev Neurosci. (2000) 1(3):199–207. 10.1038/35044563 11257908

[30] Peelen MV Downing PE. Within-subject reproducibility of category-specific visual activation with functional MRI. Hum Brain Mapp. (2005) 25(4):402–8. 10.1002/hbm.20116 15852382 PMC6871698

[31] Ter Minassian A Ricalens E Humbert S Duc F Aube C Beydon L. Dissociating anticipation from perception: acute pain activates default mode network. Hum Brain Mapp. (2013) 34(9):2228–43. 10.1002/hbm.22062 22438291 PMC6870109

[32] Veldhuijzen DS Nemenov MI Keaser M Zhuo J Gullapalli RP Greenspan JD. Differential brain activation associated with laser-evoked burning and pricking pain: an event-related fMRI study. Pain. (2009) 141(1-2):104–13. 10.1016/j.pain.2008.10.027 19058914 PMC6449044

[33] Mogenson GJ Brudzynski SM Wu M Yang CR Yim CC. From motivation to action: a review of dopaminergic regulation of limbic→ nucleus accumbens→ ventral pallidum→ pedunculopontine nucleus circuitries involved in limbic-motor integration. In: Kalivas PW, Koob GF, Barnes CD, Nemeroff CB, North RA, Seutin V, et al., editors. Limbic motor circuits and neuropsychiatry. Boca Raton: CRC Press (2019). p. 193–236.

[34] Kretschmer BD. Functional aspects of the ventral pallidum. Amino Acids. (2000) 19(1):201–10. 10.1007/s007260070050 11026490

[35] Rössler J Rössler W Seifritz E Unterrassner L Wyss T Haker H Dopamine-induced dysconnectivity between salience network and auditory cortex in subjects with psychotic-like experiences: a randomized double-blind placebo-controlled study. Schizophr Bull. (2020) 46(3):732–40. 10.1093/schbul/sbz110 31751466 PMC7147573

[36] Halligan PW Marshall JC Wade DT. Unilateral somatoparaphrenia after right hemisphere stroke: a case description. Cortex. (1995) 31(1):173–82. 10.1016/S0010-9452(13)80115-3 7781314

[37] Ben-Shabat E Matyas TA Pell GS Brodtmann A Carey LM. The right supramarginal gyrus is important for proprioception in healthy and stroke-affected participants: a functional MRI study. Front Neurol. (2015) 6:248. 10.3389/fneur.2015.00248 26696951 PMC4668288

[38] Fallon N Roberts C Stancak A. Shared and distinct functional networks for empathy and pain processing: a systematic review and meta-analysis of fMRI studies. Soc Cogn Affect Neurosci. (2020) 15(7):709–23. 10.1093/scan/nsaa090 32608498 PMC7511882

[39] Parent A Hazrati LN. Functional anatomy of the basal ganglia. I. The cortico-basal ganglia-thalamo-cortical loop. Brain Res Brain Res Rev. (1995) 20(1):91–127. 10.1016/0165-0173(94)00007-C 7711769

[40] Packard MG Knowlton BJ. Learning and memory functions of the basal ganglia. Annu Rev Neurosci. (2002) 25(1):563–93. 10.1146/annurev.neuro.25.112701.142937 12052921

[41] Yamada H Matsumoto N Kimura M. Tonically active neurons in the primate caudate nucleus and putamen differentially encode instructed motivational outcomes of action. J Neurosci. (2004) 24(14):3500–10. 10.1523/JNEUROSCI.0068-04.2004 15071097 PMC6729748

[42] Wu Z Ainsworth M Browncross H Bell AH Buckley MJ. Frontopolar cortex is a mediator of network modularity in the primate brain. bioRxiv. (2019) 1–30. 10.1101/2019.12.20.882837

[43] Wong D Dzemidzic M Talavage TM Romito LM Byrd KE. Motor control of jaw movements: an fMRI study of parafunctional clench and grind behavior. Brain Res. (2011) 1383:206–17. 10.1016/j.brainres.2011.01.096 21295015

[44] Zhao YP Ma XC Jin Z Li K Liu G Zeng YW. Cerebral activation during unilateral clenching in patients with temporomandibular joint synovitis and biting pain: an functional magnetic resonance imaging study. Chin Med J. (2011) 124(14):2136–43. 21933616

[45] Peng K Steele SC Becerra L Borsook D. Brodmann area 10: collating, integrating and high level processing of nociception and pain. Prog Neurobiol. (2018) 161:1–22. 10.1016/j.pneurobio.2017.11.004 29199137 PMC5826795

[46] Mills EP Akhter R Di Pietro F Murray GM Peck CC Macey PM Altered brainstem pain modulating circuitry functional connectivity in chronic painful temporomandibular disorder. J Pain. (2021) 22(2):219–32. 10.1016/j.jpain.2020.08.002 32896638

[47] Lin CS. Brain signature of chronic orofacial pain: a systematic review and meta-analysis on neuroimaging research of trigeminal neuropathic pain and temporomandibular joint disorders. PLoS One. (2014) 9(4):e94300. 10.1371/journal.pone.0094300 24759798 PMC3997345

[48] Ichesco E Quintero A Clauw DJ Peltier S Sundgren PM Gerstner GE Altered functional connectivity between the insula and the cingulate cortex in patients with temporomandibular disorder: a pilot study. Headache. (2012) 52(3):441–54. 10.1111/j.1526-4610.2011.01998.x 21929661 PMC3256286

[49] Zhang J Li X Jin Z Liang M Ma X. Spontaneous brain activity and connectivity in female patients with temporomandibular joint synovitis pain: a pilot functional magnetic resonance imaging study. Oral Surg Oral Med Oral Pathol Oral Radiol. (2018) 126(4):363–74. 10.1016/j.oooo.2018.04.012 30037632

[50] Oliva V Gregory R Brooks JCW Pickering AE. Central pain modulatory mechanisms of attentional analgesia are preserved in fibromyalgia. Pain. (2021) 163(1):125–36. 10.1097/j.pain.0000000000002319 PMC8675057 33941755

[51] Messina R Rocca MA Colombo B Valsasina P Meani A Falini A Dysregulation of multisensory processing stands out from an early stage of migraine: a study in pediatric patients. J Neurol. (2020) 267(3):760–9. 10.1007/s00415-019-09639-9 31745724

[52] Melzack R. Pain and the neuromatrix in the brain. J Dent Educ. (2001) 65(12):1378–82. 10.1002/j.0022-0337.2001.65.12.tb03497.x 11780656

[53] Wolfe F. Fibromyalgianess. *Arthritis Rheumatol*. (2009) 61(6):715–6. 10.1002/art.24553 19479689

[54] Brummett CM Janda AM Schueller CM Tsodikov A Morris M Williams DA Survey criteria for fibromyalgia independently predict increased postoperative opioid consumption after lower-extremity joint arthroplasty: a prospective, observational cohort study. Anesthesiology. (2013) 119(6):1434–43. 10.1097/ALN.0b013e3182a8eb1f 24343289 PMC3867739

[55] Werkman DF Carver KZ Harper DE Troost JP Aronovich S. Are outcomes of temporomandibular joint arthroscopy influenced by central sensitization? J Oral Maxillofac Surg. (2022) 80(6):980–8. 10.1016/j.joms.2022.02.009 35337769

[56] Harper DE Sayre K Schrepf A Clauw DJ Aronovich S. Impact of fibromyalgia phenotype in temporomandibular disorders. Pain Medicine. (2021) 22(9):2050–6. 10.1093/pm/pnab077 33674851 PMC8427347

[57] Pennebaker JW. The psychology of physical symptoms. In: Sanders GS Suls JM, editors. Social psychology of health and illness. New York: Psychology Press (1982). p. 99–128.

[58] Katz RS Wolfe F Michaud K. Fibromyalgia diagnosis: a comparison of clinical, survey, and American college of rheumatology criteria. Arthritis Rheum. (2006) 54(1):169–76. 10.1002/art.21533 16385512

[59] Wolfe F Clauw DJ Fitzcharles MA Goldenberg DL Hauser W Katz RS Fibromyalgia criteria and severity scales for clinical and epidemiological studies: a modification of the ACR preliminary diagnostic criteria for fibromyalgia. J Rheumatol. (2011) 38(6):1113–22. 10.3899/jrheum.100594 21285161

[60] Hollins M Harper D Gallagher S Owings EW Lim PF Miller V Perceived intensity and unpleasantness of cutaneous and auditory stimuli: an evaluation of the generalized hypervigilance hypothesis. Pain. (2009) 141(3):215–21. 10.1016/j.pain.2008.10.003 19121558 PMC2654196

[61] Galvez-Sanchez CM de la Coba P Duschek S Reyes Del Paso GA. Reliability, factor structure and predictive validity of the widespread pain Index and symptom severity scales of the 2010 American college of rheumatology criteria of fibromyalgia. J Clin Med. (2020) 9(8):2460. 10.3390/jcm9082460 PMC7464133 32752048

[62] Whitfield-Gabrieli S Nieto-Castanon A. Conn: a functional connectivity toolbox for correlated and anticorrelated brain networks. Brain Connect. (2012) 2(3):125–41. 10.1089/brain.2012.0073 22642651

[63] Whitfield-Gabrieli S Nieto-Castanon A. CONN functional connectivity toolbox. 20b ed2009. 10.1089/brain.2012.0073 22642651

[64] Dale AM Fischl B Sereno MI. Cortical surface-based analysis. I. Segmentation and surface reconstruction. Neuroimage. (1999) 9(2):179–94. 10.1006/nimg.1998.0395 9931268

[65] Fischl B Dale AM. Measuring the thickness of the human cerebral cortex from magnetic resonance images. Proc Natl Acad Sci U S A. (2000) 97(20):11050–5. 10.1073/pnas.200033797 10984517 PMC27146

[66] Fischl B Liu A Dale AM. Automated manifold surgery: constructing geometrically accurate and topologically correct models of the human cerebral cortex. IEEE Trans Med Imaging. (2001) 20(1):70–80. 10.1109/42.906426 11293693

[67] Fischl B Salat DH van der Kouwe AJ Makris N Segonne F Quinn BT Sequence-independent segmentation of magnetic resonance image. Neuroimage. (2004) 23:S69–84. 10.1016/j.neuroimage.2004.07.016 15501102

[68] Fischl B Sereno MI Dale AM. Cortical surface-based analysis. II: inflation, flattening, and a surface-based coordinate system. Neuroimage. (1999) 9(2):195–207. 10.1006/nimg.1998.0396 9931269

[69] Han X Jovicich J Salat D van der Kouwe A Quinn B Czanner S Reliability of MRI-derived measurements of human cerebral cortical thickness: the effects of field strength, scanner upgrade and manufacturer. Neuroimage. (2006) 32(1):180–94. 10.1016/j.neuroimage.2006.02.051 16651008

[70] Reuter M Rosas HD Fischl B. Highly accurate inverse consistent registration: a robust approach. Neuroimage. (2010) 53(4):1181–96. 10.1016/j.neuroimage.2010.07.020 20637289 PMC2946852

[71] Reuter M Schmansky NJ Rosas HD Fischl B. Within-subject template estimation for unbiased longitudinal image analysis. Neuroimage. (2012) 61(4):1402–18. 10.1016/j.neuroimage.2012.02.084 22430496 PMC3389460

[72] Chen Q Heinricher MM. Descending control mechanisms and chronic pain. Curr Rheumatol Rep. (2019) 21(5):13. 10.1007/s11926-019-0813-1 30830471

[73] Heinricher MM Fields HL. Central nervous system mechanisms of pain modulation. In: Koltzenburg M, McMahon SB, editors. Wall and melzack's textbook of pain. 6th ed. Philadelphia: Elsevier (2013). p. 129–42.

[74] Porreca F Ossipov MH Gebhart GF. Chronic pain and medullary descending facilitation. Trends Neurosci. (2002) 25(6):319–25. 10.1016/S0166-2236(02)02157-4 12086751

[75] Ren K Dubner R. Descending modulation in persistent pain: an update. Pain. (2002) 100(1-2):1–6. 10.1016/S0304-3959(02)00368-8 12435453

[76] Taylor KS Seminowicz DA Davis KD. Two systems of resting state connectivity between the insula and cingulate cortex. Hum Brain Mapp. (2009) 30(9):2731–45. 10.1002/hbm.20705 19072897 PMC6871122

[77] Iannetti GD Mouraux A. From the neuromatrix to the pain matrix (and back). Exp Brain Res. (2010) 205(1):1–12. 10.1007/s00221-010-2340-1 20607220

[78] Makris N Goldstein JM Kennedy D Hodge SM Caviness VS Faraone SV Decreased volume of left and total anterior insular lobule in schizophrenia. Schizophr Res. (2006) 83(2-3):155–71. 10.1016/j.schres.2005.11.020 16448806

[79] Frazier JA Chiu S Breeze JL Makris N Lange N Kennedy DN Structural brain magnetic resonance imaging of limbic and thalamic volumes in pediatric bipolar disorder. Am J Psychiatry. (2005) 162(7):1256–65. 10.1176/appi.ajp.162.7.1256 15994707

[80] Desikan RS Ségonne F Fischl B Quinn BT Dickerson BC Blacker D An automated labeling system for subdividing the human cerebral cortex on MRI scans into gyral based regions of interest. Neuroimage. (2006) 31(3):968–80. 10.1016/j.neuroimage.2006.01.021 16530430

[81] Tzourio-Mazoyer N Landeau B Papathanassiou D Crivello F Etard O Delcroix N Automated anatomical labeling of activations in SPM using a macroscopic anatomical parcellation of the MNI MRI single-subject brain. Neuroimage. (2002) 15(1):273–89. 10.1006/nimg.2001.0978 11771995

[82] Diedrichsen J. A spatially unbiased atlas template of the human cerebellum. Neuroimage. (2006) 33(1):127–38. 10.1016/j.neuroimage.2006.05.056 16904911

[83] Diedrichsen J Balsters JH Flavell J Cussans E Ramnani N. A probabilistic MR atlas of the human cerebellum. Neuroimage. (2009) 46(1):39–46. 10.1016/j.neuroimage.2009.01.045 19457380

[84] Diedrichsen J Hernaandez-Castillo C King M Prichard G Lally N Wiestler J. SUIT toolbox. 3.4 ed2006.

[85] Diedrichsen J Maderwald S Kuper M Thurling M Rabe K Gizewski ER Imaging the deep cerebellar nuclei: a probabilistic atlas and normalization procedure. Neuroimage. (2011) 54(3):1786–94. 10.1016/j.neuroimage.2010.10.035 20965257

[86] Diedrichsen J Zotow E. Surface-based display of volume-averaged cerebellar imaging data. PLoS One. (2015) 10(7):e0133402. 10.1371/journal.pone.0133402 26230510 PMC4521932

[87] Coulombe MA Lawrence KS Moulin DE Morley-Forster P Shokouhi M Nielson WR Lower functional connectivity of the periaqueductal gray is related to negative affect and clinical manifestations of fibromyalgia. Front Neuroanat. (2017) 11:47. 10.3389/fnana.2017.00047 28642688 PMC5462926

[88] Li P Shan H Nie B Liu H Dong G Guo Y Sleeve gastrectomy rescuing the altered functional connectivity of lateral but not medial hypothalamus in subjects with obesity. Obes Surg. (2019) 29(7):2191–9. 10.1007/s11695-019-03822-7 30895508

[89] Bullmore ET Suckling J Overmeyer S Rabe-Hesketh S Taylor E Brammer MJ. Global, voxel, and cluster tests, by theory and permutation, for a difference between two groups of structural MR images of the brain. IEEE Trans Med Imaging. (1999) 18(1):32–42. 10.1109/42.750253 10193695

[90] Nichols TE Holmes AP. Nonparametric permutation tests for functional neuroimaging: a primer with examples. Hum Brain Mapp. (2002) 15(1):1–25. 10.1002/hbm.1058 11747097 PMC6871862

[91] Hayasaka S Nichols TE. Combining voxel intensity and cluster extent with permutation test framework. Neuroimage. (2004) 23(1):54–63. 10.1016/j.neuroimage.2004.04.035 15325352

[92] Zalesky A Fornito A Bullmore ET. Network-based statistic: identifying differences in brain networks. Neuroimage. (2010) 53(4):1197–207. 10.1016/j.neuroimage.2010.06.041 20600983

[93] Zalesky A Fornito A Bullmore E. On the use of correlation as a measure of network connectivity. Neuroimage. (2012) 60(4):2096–106. 10.1016/j.neuroimage.2012.02.001 22343126

[94] Nieto-Castanon A. Handbook of functional connectivity magnetic resonance imaging methods in CONN. Boston: Hilbert Press (2020).

[95] Rubinov M Sporns O. Complex network measures of brain connectivity: uses and interpretations. Neuroimage. (2010) 52(3):1059–69. 10.1016/j.neuroimage.2009.10.003 19819337

[96] Watts DJ Strogatz SH. Collective dynamics of ‘small-world’ networks. Nature. (1998) 393(6684):440–2. 10.1038/30918 9623998

[97] Achard S Bullmore E. Efficiency and cost of economical brain functional networks. PLoS Comput Biol. (2007) 3(2):e17. 10.1371/journal.pcbi.0030017 17274684 PMC1794324

[98] Latora V Marchiori M. Efficient behavior of small-world networks. Phys Rev Lett. (2001) 87(19):198701. 10.1103/PhysRevLett.87.198701 11690461

[99] Blondel VD Guillaume J-L Lambiotte R Lefebvre E. Fast unfolding of communities in large networks. J Stat Mech: Theory Exp. (2008) 2008(10):P10008. 10.1088/1742-5468/2008/10/P10008

[100] Seabold S Perktold J. Statsmodels: econometric and statistical modeling with python. Proceedings of the 9th python in science conference. Austin, TX (2010).

[101] Lindstrom MJ Bates DM. Newton—raphson and EM algorithms for linear mixed-effects models for repeated-measures data. J Am Stat Assoc. (1988) 83(404):1014–22. 10.1080/01621459.1988.10478693

[102] Gustin SM Peck CC Cheney LB Macey PM Murray GM Henderson LA. Pain and plasticity: is chronic pain always associated with somatosensory cortex activity and reorganization? J Neurosci. (2012) 32(43):14874–84. 10.1523/JNEUROSCI.1733-12.2012 23100410 PMC6704842

[103] Moayedi M Weissman-Fogel I Salomons TV Crawley AP Goldberg MB Freeman BV White matter brain and trigeminal nerve abnormalities in temporomandibular disorder. Pain. (2012) 153(7):1467–77. 10.1016/j.pain.2012.04.003 22647428

[104] Chen Z Liu M Wang B Fan W Zhang X Hu M Evaluation of brain volume changes in patients with painful temporomandibular disorders using voxel-based morphometry. Zhonghua Kou Qiang Yi Xue Za Zhi. (2020) 55(9):624–8. 32878396 10.3760/cma.j.cn112144-20200514-00273

[105] Domin M Grimm NK Klepzig K Schmidt CO Kordass B Lotze M. Gray matter brain alterations in temporomandibular disorder tested in a population cohort and three clinical samples. J Pain. (2021) 22(6):739–47. 10.1016/j.jpain.2021.01.003 33529707

[106] Moayedi M Weissman-Fogel I Crawley AP Goldberg MB Freeman BV Tenenbaum HC Contribution of chronic pain and neuroticism to abnormal forebrain gray matter in patients with temporomandibular disorder. Neuroimage. (2011) 55(1):277–86. 10.1016/j.neuroimage.2010.12.013 21156210

[107] Yelnik J. Functional anatomy of the basal ganglia. Mov Disord. (2002) 17(Suppl 3):S15–21. 10.1002/mds.10138 11948751

[108] Grillner S Hellgren J Menard A Saitoh K Wikström MA. Mechanisms for selection of basic motor programs–roles for the striatum and pallidum. Trends Neurosci. (2005) 28(7):364–70. 10.1016/j.tins.2005.05.004 15935487

[109] Zhang G Ma J Lu W Zhan H Zhang X Wang K Comorbid depressive symptoms can aggravate the functional changes of the pain matrix in patients with chronic back pain: a resting-state fMRI study. Front Aging Neurosci. (2022) 14:795. 10.3389/fnagi.2022.935242 PMC9340779 35923542

[110] Letzen JE Boissoneault J Sevel LS Robinson ME. Altered mesocorticolimbic functional connectivity in chronic low back pain patients at rest and following sad mood induction. Brain Imaging Behav. (2020) 14(4):1118–29. 10.1007/s11682-019-00076-w 30877469 PMC6745297

[111] Hadjistavropoulos HD Hadjistavropoulos T Quine A. Health anxiety moderates the effects of distraction versus attention to pain. Behav Res Ther. (2000) 38(5):425–38. 10.1016/S0005-7967(99)00044-3 10816903

[112] Pincus T Morley S. Cognitive-processing bias in chronic pain: a review and integration. Psychol Bull. (2001) 127(5):599. 10.1037/0033-2909.127.5.599 11548969

[113] Kutch JJ Ichesco E Hampson JP Labus JS Farmer MA Martucci KT Brain signature and functional impact of centralized pain: a multidisciplinary approach to the study of chronic pelvic pain (MAPP) network study. Pain. (2017) 158(10):1979. 10.1097/j.pain.0000000000001001 28692006 PMC5964335

[114] Wiech K Lin C-S Brodersen KH Bingel U Ploner M Tracey I. Anterior insula integrates information about salience into perceptual decisions about pain. J Neurosci. (2010) 30(48):16324–31. 10.1523/JNEUROSCI.2087-10.2010 21123578 PMC6634837

[115] Brown CA Seymour B El-Deredy W Jones AK. Confidence in beliefs about pain predicts expectancy effects on pain perception and anticipatory processing in right anterior insula. Pain. (2008) 139(2):324–32. 10.1016/j.pain.2008.04.028 18584963

[116] Seymour B O'Doherty JP Dayan P Koltzenburg M Jones AK Dolan RJ Temporal difference models describe higher-order learning in humans. Nature. (2004) 429(6992):664–7. 10.1038/nature02581 15190354

[117] Lutz A McFarlin DR Perlman DM Salomons TV Davidson RJ. Altered anterior insula activation during anticipation and experience of painful stimuli in expert meditators. Neuroimage. (2013) 64:538–46. 10.1016/j.neuroimage.2012.09.030 23000783 PMC3787201

[118] Buvanendran A Ali A Stoub TR Berger RA Kroin JS. The use of brain positron emission tomography to identify sites of postoperative pain processing with and without epidural analgesia. Anesth Analg. (2007) 105(6):1784–6. 10.1213/01.ane.0000270206.30333.cb 18042883

[119] Becker S Gandhi W Pomares F Wager TD Schweinhardt P. Orbitofrontal cortex mediates pain inhibition by monetary reward. Soc Cogn Affect Neurosci. (2017) 12(4):651–61. 10.1093/scan/nsw173 28119505 PMC5390724

[120] Liu J Zhao L Lei F Zhang Y Yuan K Gong Q Disrupted resting-state functional connectivity and its changing trend in migraine suffers. Hum Brain Mapp. (2015) 36(5):1892–907. 10.1002/hbm.22744 25640857 PMC6869678

[121] Lamichhane B Jayasekera D Jakes R Ray WZ Leuthardt EC Hawasli AH. Functional disruptions of the brain in low back pain: a potential imaging biomarker of functional disability. Front Neurol. (2021) 12:669076. 10.3389/fneur.2021.669076 34335444 PMC8317987

[122] Huang S Wakaizumi K Wu B Shen B Wu B Fan L Whole-brain functional network disruption in chronic pain with disc herniation. Pain. (2019) 160(12):2829. 10.1097/j.pain.0000000000001674 31408051 PMC6856436

[123] Mano H Kotecha G Leibnitz K Matsubara T Sprenger C Nakae A Classification and characterisation of brain network changes in chronic back pain: a multicenter study. Wellcome Open Res. (2018) 3:19. 10.12688/wellcomeopenres.14069.2 29774244 PMC5930551

[124] De Pauw R Aerts H Siugzdaite R Meeus M Coppieters I Caeyenberghs K Hub disruption in patients with chronic neck pain: a graph analytical approach. Pain. (2020) 161(4):729–41. 10.1097/j.pain.0000000000001762 31764388

[125] Komaki Y Hikishima K Shibata S Konomi T Seki F Yamada M Functional brain mapping using specific sensory-circuit stimulation and a theoretical graph network analysis in mice with neuropathic allodynia. Sci Rep. (2016) 6(1):1–11. 10.1038/srep37802 27898057 PMC5127182

[126] Bilbao A Falfan-Melgoza C Leixner S Becker R Singaravelu SK Sack M Longitudinal structural and functional brain network alterations in a mouse model of neuropathic pain. Neuroscience. (2018) 387:104–15. 10.1016/j.neuroscience.2018.04.020 29694917

[127] Imai N. Altered occipital pole connectivity in chronic versus episodic migraine: whole brain region-of-interest analysis of resting-state functional connectivity. Neurol Clin Neurosci. (2018) 6(6):173–8. 10.1111/ncn3.12229

[128] Kurokawa R Kamiya K Inui S Kato S Suzuki F Amemiya S Structural connectivity changes in the cerebral pain matrix in burning mouth syndrome: a multi-shell, multi-tissue-constrained spherical deconvolution model analysis. Neuroradiology. (2021) 63(12):2005–12. 10.1007/s00234-021-02732-9 34142212

[129] Labus JS Dinov ID Jiang Z Ashe-McNalley C Zamanyan A Shi Y Irritable bowel syndrome in female patients is associated with alterations in structural brain networks. Pain. (2014) 155(1):137–49. 10.1016/j.pain.2013.09.020 24076048 PMC4100785

[130] He S-S Li F Song F Wu S Chen J-Y He N Spontaneous neural activity alterations in temporomandibular disorders: a cross-sectional and longitudinal resting-state functional magnetic resonance imaging study. Neuroscience. (2014) 278:1–10. 10.1016/j.neuroscience.2014.07.067 25110816

[131] Marek S Tervo-Clemmens B Calabro FJ Montez DF Kay BP Hatoum AS Reproducible brain-wide association studies require thousands of individuals. Nature. (2022) 603(7902):654–60. 10.1038/s41586-022-04492-9 35296861 PMC8991999

[132] Napadow V LaCount L Park K As-Sanie S Clauw DJ Harris RE. Intrinsic brain connectivity in fibromyalgia is associated with chronic pain intensity. Arthritis Rheum. (2010) 62(8):2545–55. 10.1002/art.27497 20506181 PMC2921024

[133] Čeko M Frangos E Gracely J Richards E Wang B Schweinhardt P Default mode network changes in fibromyalgia patients are largely dependent on current clinical pain. Neuroimage. (2020) 216:116877. 10.1016/j.neuroimage.2020.116877 32344063 PMC8855626

