# Supplementary material for: Topology of pain networks in patients with temporomandibular disorder and pain-free controls with and without concurrent experimental pain: A pilot study
Source: Front Pain Res (Lausanne). 2022 Oct 17;3:966398. doi: 10.3389/fpain.2022.966398 (PMC9619074; doi:10.3389/fpain.2022.966398)
Supplement: Supplementary file 1 [file DataSheet1.docx]

**Supplementary Methods**

## Additional details on MRI preprocessing

The CONN minimal preprocessing pipeline wraps SPM12 (Functional Imaging Laboratory (FIL) and Wellcome Center for Human Neuroimaging, 2014) functions, and consists of five steps. First, rsfMRI data is subjected to the SPM12 ‘Realign and Unwarp’ procedure, which co-registers all participants’ rsfMRI acquisitions to the first scan of their first series (here, the first TR of the cuff-deflated condition) using *b-*splines and estimates the derivatives of the deformation field with respect to head movement, thereby minimizing data variance due to the susceptibility-by-motion interaction while retaining as much of the experimentally-induced variance as possible (Andersson et al., 2001). Second, the pipeline applies standard motion and slice timing correction for multiband acquisitions using SPM12 (Henson et al., 1999; Woletz et al., 2014). Third, potential outlier scans for each participant, defined as framewise displacements greater than 0.9mm or global BOLD signal differences greater than 5 standard deviations from the participant’s mean, are ‘flagged’ for further analysis using Artifact Detection Tools (ART: Mozes and Whitfield-Gabrieli, 2015). Fourth, rsfMRI and T1-anatomical data are normalized to MNI152 stereotactic space, and grey matter, white matter, and cerebrospinal fluid posterior tissue maps estimated via the SPM12 ‘Unified Segmentation and Normalization’ procedure (Ashburner and Friston, 2005). This procedure also reduces the geometric distortions in the rsfMRI EPI images due to susceptibility artefacts using nonlinear registration of the fieldmaps (Calhoun et al., 2017) Finally, rsfMRI data is smoothed using a Gaussian kernel with an 8mm filter width half maximum to increase signal-to-noise ratio and reduce residual variability due to inter-subject differences in functional and gyral anatomy.

The CONN denoising pipeline consists of estimation and removal of potential confound effects from participants’ BOLD timeseries using an anatomical component-based noise correction procedure, aCompCor (Behzadi et al., 2007), as well as temporal bandpass filtering. These confounds include the average BOLD signal from white matter (derived from voxels with values above 50% in white matter, followed by a 1-voxel binary erosion step) and cerebrospinal fluid posterior probability maps; the first four principal components within both of these compartments (derived from a principal components analysis of the covariance within the subspace orthogonal to the average BOLD signal and all other potential confound effects); translation and rotation parameters and their first-order derivatives; ‘flagged’ outlier scans (TRs); and constant and linear BOLD signal trends within each participant’s imaging series (i.e., cuff-deflated and cuff-inflated acquisitions), which presumably represent slow BOLD signal trends and initial magnetization transients, and are convolved with a canonical hemodynamic response function (Chai et al., 2012; Friston et al., 1996; Power et al., 2014; Behzadi et al., 2007; Whitfield-Gabrieli and Nieto-Castanon, 2012). Confound effects are estimated and removed separately for each voxel, participant, and imaging series using ordinary least-squares regression (Whitfield-Gabrieli and Nieto-Castanon, 2012). Residual data is then lowpass-filtered at 250mHz using discrete cosine transform windowing (Birn et al., 2006; Fox et al., 2005; Fox et al., 2009).

## Graph theory analyses

Graph-theoretic metrics for this study included nodal degree, betweenness centrality, and community. For unweighted, undirected networks such as those defined by the present study’s four adjacency matrices, the *nodal degree* is computed as the sum of suprathreshold connections between a given node $k$ and all other nodes $i$, relative to the number of nodes $N$ in the entire network (after applying some threshold criterion):

$$d_{k}=\sum_{i\in N} a_{ik}$$

Where $a_{ij}$ represents the connection weight for a given connection between nodes $i$ and $k$, and when drawn from a binary adjacency matrix is either 0 or 1. Consequently, a node’s degree simply serves as a measure of local integration (Rubinov and Sporns, 2010).

The *betweenness centrality* is defined as the fraction of shortest network-spanning paths passing through a node. If the number of shortest paths between two nodes $i$ and $j$ is denoted $\rho_{ij}$, some fraction of these paths $\rho_{ij}(k)$ passes through a given node *k* which is connected to them:

$$b_{k}=\sum_{i\neq k\neq j} \frac{\rho_{ij}\left( k \right)}{\rho_{ij}}$$

In practice, this metric is normalized to the total number of node pairs which do not include $k$. For undirected graphs such as those represented by, the normalization term is as follows:

$$c_{k}=\frac{1}{\left( n-1 \right)\left( n-2 \right)}b_{k}$$

High betweenness centralities therefore represent nodes which ‘bridge’ or link subsections of a network (Rubinov and Sporns, 2010).

The Louvain algorithm and its outputs can be viewed as an iterative, hierarchical partitioning of a network into subgraphs. Given two nodes $i$ and $j$, a ‘modularity’ estimate $Q$ is computed using the connection weights $a_{ij}$ between them, the sum of the weights of all suprathreshold connections with node $i$ ($a_{i}$) and with node $j$ ($a_{j})$, and the sum of all suprathreshold weights in the network, $m$:

$$Q=\frac{1}{2m}\sum_{i,j} \left[ a_{ij}-\frac{a_{i}a_{j}}{2m} \right]\delta(c_{i}{,c}_{j})$$

The Louvain algorithm proceeds by building ‘local’ communities among individual nodes by computing the gain $\Delta Q$ in each node’s modularity with each merge. It then partitions a ‘new’ network using the local communities as ‘nodes.’ This process is repeated at higher (more abstracted) levels of the network hierarchy until a maximum modularity is attained (Blondel et al., 2008).

**References**

Andersson, J. L., Hutton, C., Ashburner, J., Turner, R. & Friston, K. 2001. Modeling geometric deformations in EPI time series. *Neuroimage,* 13(5)**,** pp 903-19.

Ashburner, J. & Friston, K. J. 2005. Unified segmentation. *Neuroimage,* 26(3)**,** pp 839-51.

Behzadi, Y., Restom, K., Liau, J. & Liu, T. T. 2007. A component based noise correction method (CompCor) for BOLD and perfusion based fMRI. *Neuroimage,* 37(1)**,** pp 90-101.

Birn, R. M., Diamond, J. B., Smith, M. A. & Bandettini, P. A. 2006. Separating respiratory-variation-related fluctuations from neuronal-activity-related fluctuations in fMRI. *Neuroimage,* 31(4)**,** pp 1536-48.

Blondel, V. D., Guillaume, J.-L., Lambiotte, R. & Lefebvre, E. 2008. Fast unfolding of communities in large networks. *Journal of statistical mechanics: theory and experiment,* 2008(10)**,** pp P10008.

Calhoun, V. D., Wager, T. D., Krishnan, A., Rosch, K. S., Seymour, K. E., Nebel, M. B., Mostofsky, S. H., Nyalakanai, P. & Kiehl, K. 2017. The impact of T1 versus EPI spatial normalization templates for fMRI data analyses. *Hum Brain Mapp,* 38(11)**,** pp 5331-5342.

Chai, X. J., Castañón, A. N., Öngür, D. & Whitfield-Gabrieli, S. 2012. Anticorrelations in resting state networks without global signal regression. *Neuroimage,* 59(2)**,** pp 1420-1428.

Fox, M. D., Snyder, A. Z., McAvoy, M. P., Barch, D. M. & Raichle, M. E. 2005. The BOLD onset transient: identification of novel functional differences in schizophrenia. *Neuroimage,* 25(3)**,** pp 771-82.

Fox, M. D., Zhang, D., Snyder, A. Z. & Raichle, M. E. 2009. The global signal and observed anticorrelated resting state brain networks. *J Neurophysiol,* 101(6)**,** pp 3270-83.

Friston, K. J., Williams, S., Howard, R., Frackowiak, R. S. & Turner, R. 1996. Movement-related effects in fMRI time-series. *Magn Reson Med,* 35(3)**,** pp 346-55.

Functional Imaging Laboratory (FIL) and Wellcome Center for Human Neuroimaging, U. C. L. 2014. Statistical Parametric Mapping (SPM12). 2020 January 13 ed.

Henson, R., Buechel, C., Josephs, O. & Friston, K. 1999. The slice-timing problem in event-related fMRI. *NeuroImage,* 9(125-.

Mozes, S. & Whitfield-Gabrieli, S. 2015. Artifact Detection Tools (ART). 2015.10 ed.

Power, J. D., Mitra, A., Laumann, T. O., Snyder, A. Z., Schlaggar, B. L. & Petersen, S. E. 2014. Methods to detect, characterize, and remove motion artifact in resting state fMRI. *Neuroimage,* 84(320-41.

Rubinov, M. & Sporns, O. 2010. Complex network measures of brain connectivity: uses and interpretations. *Neuroimage,* 52(3)**,** pp 1059-69.

Whitfield-Gabrieli, S. & Nieto-Castanon, A. 2012. Conn: a functional connectivity toolbox for correlated and anticorrelated brain networks. *Brain Connect,* 2(3)**,** pp 125-41.

Woletz, M., Hoffmann, A., Ganger, S., Paul, K., Seiger, R., Pfabigan, D., Hahn, A., Sladky, R., Lamm, C. & Lanzenberger, R. Slice-timing correction for multi-band images in SPM. 20th Meeting of the Organization for Human Brain Mapping, HBM‐Hamburg, 2014.
