# Supplementary material for: Topology of pain networks in patients with temporomandibular disorder and pain-free controls with and without concurrent experimental pain: A pilot study
Source: Front Pain Res (Lausanne). 2022 Oct 17;3:966398. doi: 10.3389/fpain.2022.966398 (PMC9619074; doi:10.3389/fpain.2022.966398)
Supplement: Supplementary file 2 [file DataSheet2.docx]

**Supplementary Methods**

## Additional details on MRI preprocessing

The CONN minimal preprocessing pipeline wraps SPM12 {Functional Imaging Laboratory (FIL) and Wellcome Center for Human Neuroimaging, 2014 #1480} functions, and consists of five steps. First, rsfMRI data is subjected to the SPM12 ‘Realign and Unwarp’ procedure, which co-registers all participants’ rsfMRI acquisitions to the first scan of their first series (here, the first TR of the cuff-deflated condition) using *b-*splines and estimates the derivatives of the deformation field with respect to head movement, thereby minimizing data variance due to the susceptibility-by-motion interaction while retaining as much of the experimentally-induced variance as possible {Andersson, 2001 #1475}. Second, the pipeline applies standard motion and slice timing correction for multiband acquisitions using SPM12 {Henson, 1999 #1477;Woletz, 2014 #1478}. Third, potential outlier scans for each participant, defined as framewise displacements greater than 0.9mm or global BOLD signal differences greater than 5 standard deviations from the participant’s mean, are ‘flagged’ for further analysis using Artifact Detection Tools {ART: \Mozes, 2015 #1479}. Fourth, rsfMRI and T1-anatomical data are normalized to MNI152 stereotactic space, and grey matter, white matter, and cerebrospinal fluid posterior tissue maps estimated via the SPM12 ‘Unified Segmentation and Normalization’ procedure {Ashburner, 2005 #1476}. This procedure also reduces the geometric distortions in the rsfMRI EPI images due to susceptibility artefacts using nonlinear registration of the fieldmaps {Calhoun, 2017 #1459} Finally, rsfMRI data is smoothed using a Gaussian kernel with an 8mm filter width half maximum to increase signal-to-noise ratio and reduce residual variability due to inter-subject differences in functional and gyral anatomy.

The CONN denoising pipeline consists of estimation and removal of potential confound effects from participants’ BOLD timeseries using an anatomical component-based noise correction procedure, aCompCor {Behzadi, 2007 #125}, as well as temporal bandpass filtering. These confounds include the average BOLD signal from white matter (derived from voxels with values above 50% in white matter, followed by a 1-voxel binary erosion step) and cerebrospinal fluid posterior probability maps; the first four principal components within both of these compartments (derived from a principal components analysis of the covariance within the subspace orthogonal to the average BOLD signal and all other potential confound effects); translation and rotation parameters and their first-order derivatives; ‘flagged’ outlier scans (TRs); and constant and linear BOLD signal trends within each participant’s imaging series (i.e., cuff-deflated and cuff-inflated acquisitions), which presumably represent slow BOLD signal trends and initial magnetization transients, and are convolved with a canonical hemodynamic response function {Chai, 2012 #233;Friston, 1996 #1482;Power, 2014 #1483;Behzadi, 2007 #125;Whitfield-Gabrieli, 2012 #138}. Confound effects are estimated and removed separately for each voxel, participant, and imaging series using ordinary least-squares regression {Whitfield-Gabrieli, 2012 #138}. Residual data is then lowpass-filtered at 250mHz using discrete cosine transform windowing {Birn, 2006 #140;Fox, 2005 #139;Fox, 2009 #141}.

## Graph theory analyses

Graph-theoretic metrics for this study included nodal degree, betweenness centrality, and community. For unweighted, undirected networks such as those defined by the present study’s four adjacency matrices, the *nodal degree* is computed as the sum of suprathreshold connections between a given node $k$ and all other nodes $i$, relative to the number of nodes $N$ in the entire network (after applying some threshold criterion):

$$d_{k}=\sum_{i\in N} a_{ik}$$

Where $a_{ij}$ represents the connection weight for a given connection between nodes $i$ and $k$, and when drawn from a binary adjacency matrix is either 0 or 1. Consequently, a node’s degree simply serves as a measure of local integration {Rubinov 2010}.

The *betweenness centrality* is defined as the fraction of shortest network-spanning paths passing through a node. If the number of shortest paths between two nodes $i$ and $j$ is denoted $\rho_{ij}$, some fraction of these paths $\rho_{ij}(k)$ passes through a given node *k* which is connected to them:

$$b_{k}=\sum_{i\neq k\neq j} \frac{\rho_{ij}\left( k \right)}{\rho_{ij}}$$

In practice, this metric is normalized to the total number of node pairs which do not include $k$. For undirected graphs such as those represented by, the normalization term is as follows:

$$c_{k}=\frac{1}{\left( n-1 \right)\left( n-2 \right)}b_{k}$$

High betweenness centralities therefore represent nodes which ‘bridge’ or link subsections of a network {Rubinov, 2010 #1050}.

The Louvain algorithm and its outputs can be viewed as an iterative, hierarchical partitioning of a network into subgraphs. Given two nodes $i$ and $j$, a ‘modularity’ estimate $Q$ is computed using the connection weights $a_{ij}$ between them, the sum of the weights of all suprathreshold connections with node $i$ ($a_{i}$) and with node $j$ ($a_{j})$, and the sum of all suprathreshold weights in the network, $m$:

$$Q=\frac{1}{2m}\sum_{i,j} \left[ a_{ij}-\frac{a_{i}a_{j}}{2m} \right]\delta(c_{i}{,c}_{j})$$

The Louvain algorithm proceeds by building ‘local’ communities among individual nodes by computing the gain $\Delta Q$ in each node’s modularity with each merge. It then partitions a ‘new’ network using the local communities as ‘nodes.’ This process is repeated at higher (more abstracted) levels of the network hierarchy until a maximum modularity is attained {Blondel, 2008 #1500}.
