# Supplementary material for: Topology of pain networks in patients with temporomandibular disorder and pain-free controls with and without concurrent experimental pain: A pilot study
Source: Front Pain Res (Lausanne). 2022 Oct 17;3:966398. doi: 10.3389/fpain.2022.966398 (PMC9619074; doi:10.3389/fpain.2022.966398)
Supplement: Supplementary file 3 [file Image1.pdf]

Supplementary Figures

QUALITY ASSURANCE

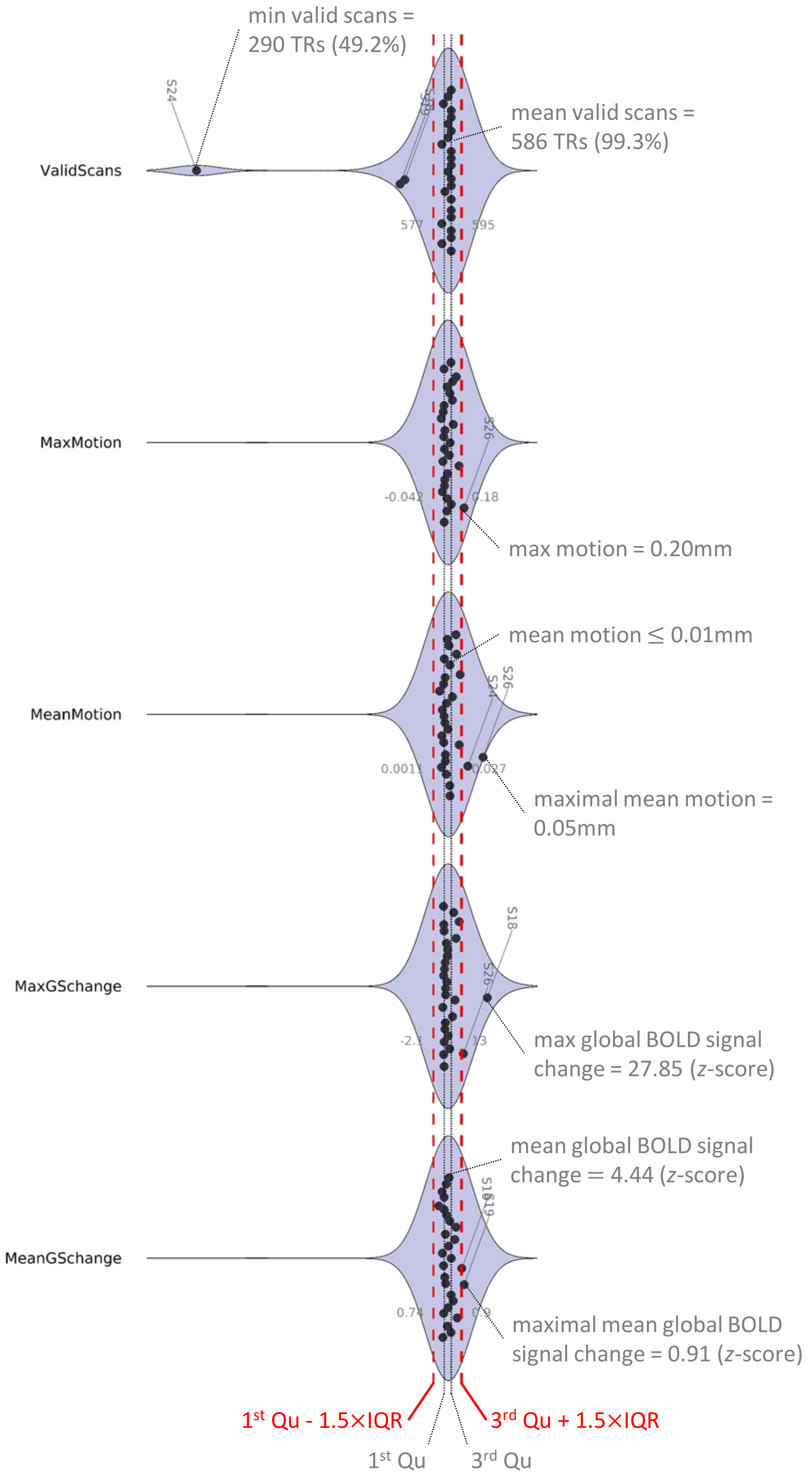

Supplementary Figure 1. Quality control and assurance results from the aCompCor denoising workflow, as implemented in the CONN Toolbox. This figure depicts the distributions of the number of valid scans (*ValidScans*) after denoising, mean and maximal framewise motion (*MeanMotion*, *MaxMotion*), and mean and maximal framewise global signal change (*MeanGSchange*, *MaxGCchange*). Thin grey lines represent first and third quartiles (*1st Qu*, *3rd Qu*) for each metric, and dashed red lines the first or third quartile plus or minus 1.5 times the interquartile range (*IQR*). Mean and maximal framewise displacement prior to denoising were less than 0.01mm and 0.20mm, respectively, meaning that the maximum framewise motion was less than 0.08% of the smallest voxel dimension, 2.386mm. The CONN Toolbox depicts global BOLD signal change in *z*-scores; the mean and maximal global signal change prior to denoising were *z* = 4.44 and 27.85, respectively. TR-to-TR displacements > 0.90mm and global signal changes > 5 standard deviations are flagged as outliers and excluded from further analysis. The minimal number of valid TRs after the denoising workflow, 290 TRs or 49.2% of the total number of acquisitions, was observed in one TMD participant, S24, who exhibited comparatively higher degrees of framewise motion over the scanning interval (mean motion = 0.03mm) and a moderate degree of mean global signal change (*z* = 0.90), and was therefore subject to TR exclusion based on the 0.90mm/5SD criteria. Most scans were retained across participants, with a mean number of valid scans = 586 of 590 TRs, or 99.3% of the total acquisition.

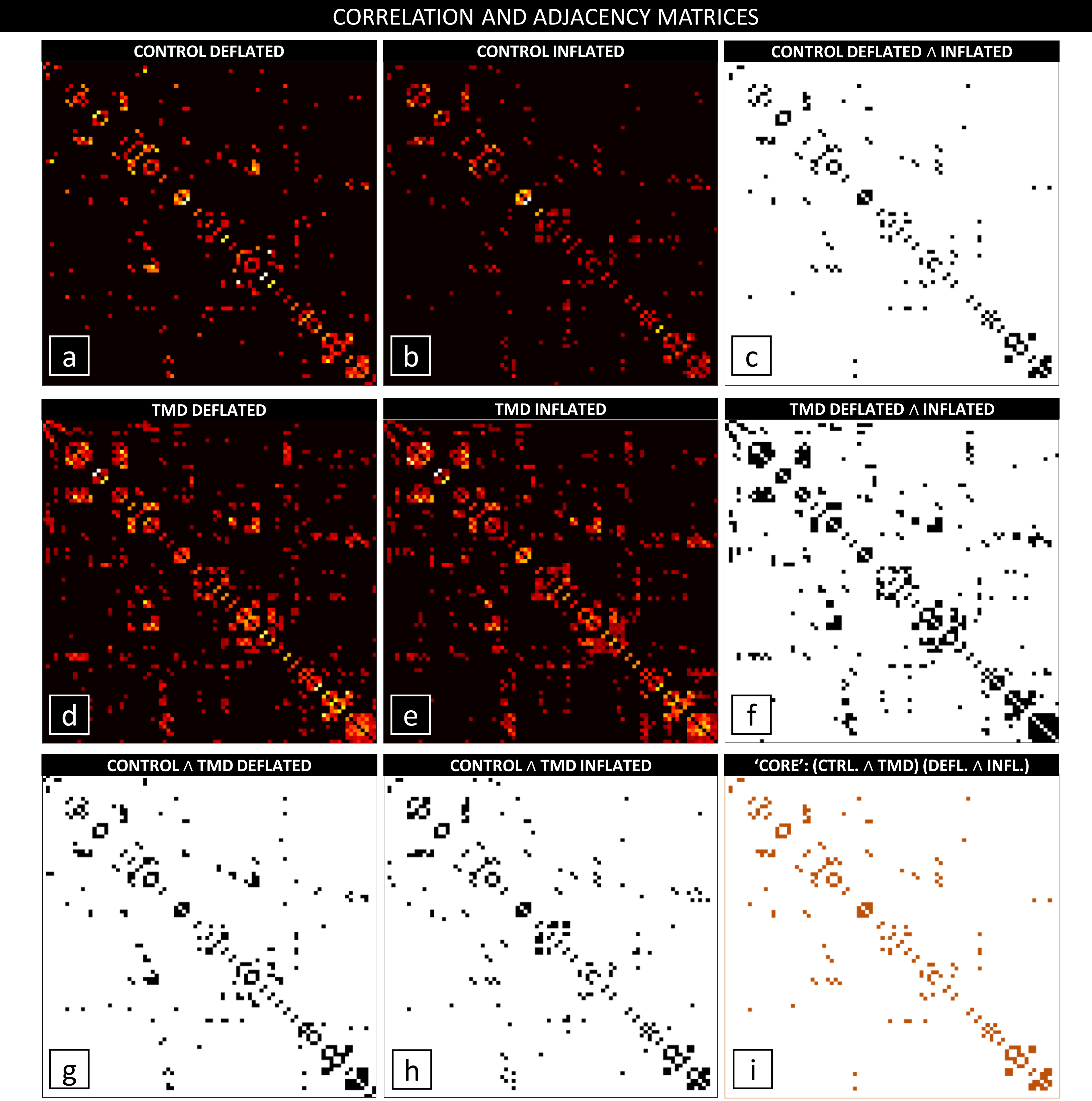

Supplementary Figure 2. Correlation (panels a, b, d, and e) and adjacency matrices (panels c, f, and g-i) for subject groups and cuff conditions. Correlation matrices for control participants under the cuff-deflated and cuff-inflated conditions in panels (a) and (b), respectively, and for TMD participants in panels (d) and (e), respectively. Connections surviving the one-sample *t*-test in each condition were assigned a value of 1 in the control-inflated, control-deflated, TMD-inflated, and TMD-deflated adjacency matrices. An elementwise multiplication of these matrices (AND operation, ∧) produced secondary adjacency matrices representing connections common to both conditions in control (c) or TMD participants (f), or connections common to both groups in the deflated (g) or inflated (h) condition. Finally, connections which survived one-sample *t*-tests across both groups and both conditions were considered to constitute a 'core' network, shown in panel (i).

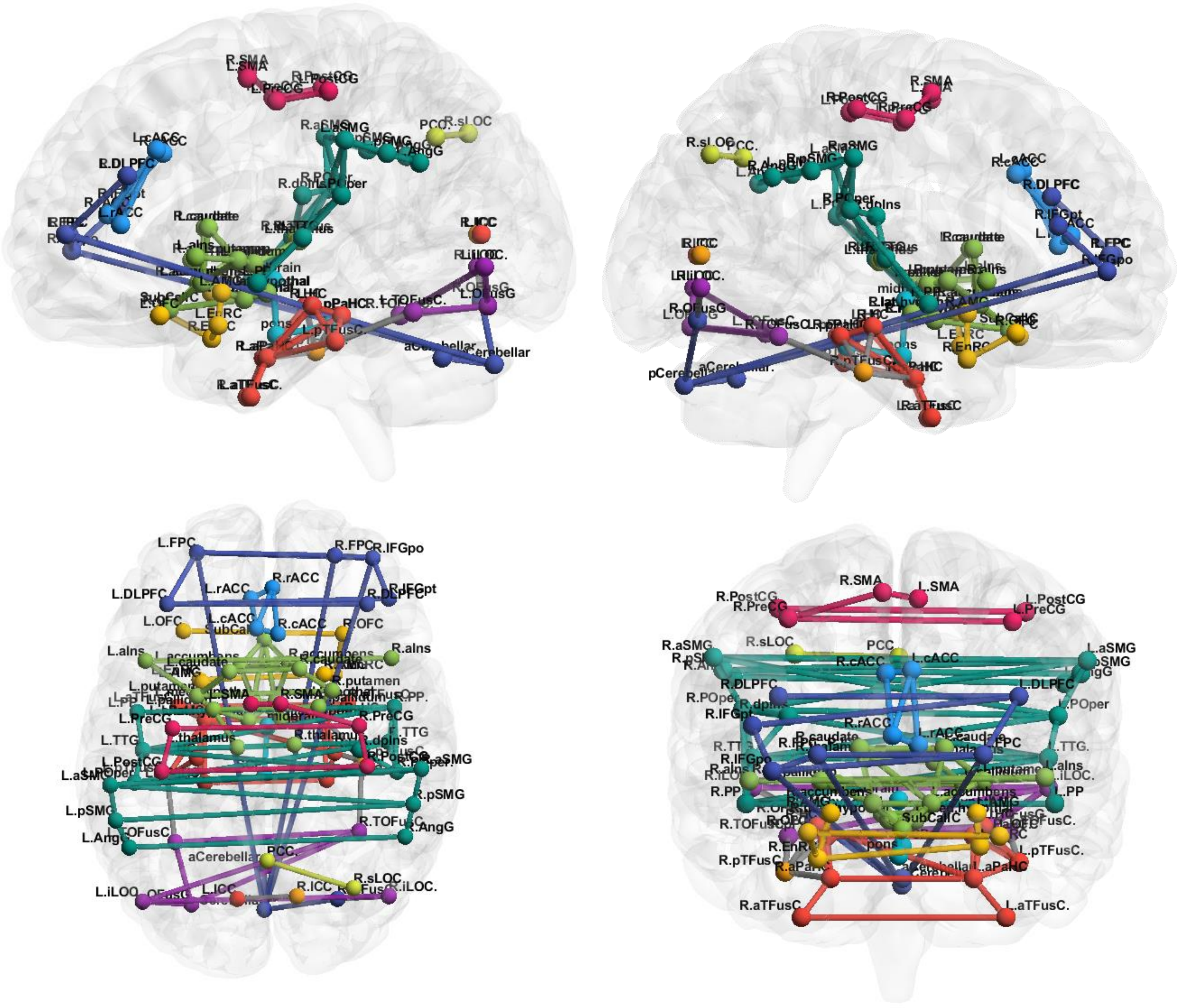

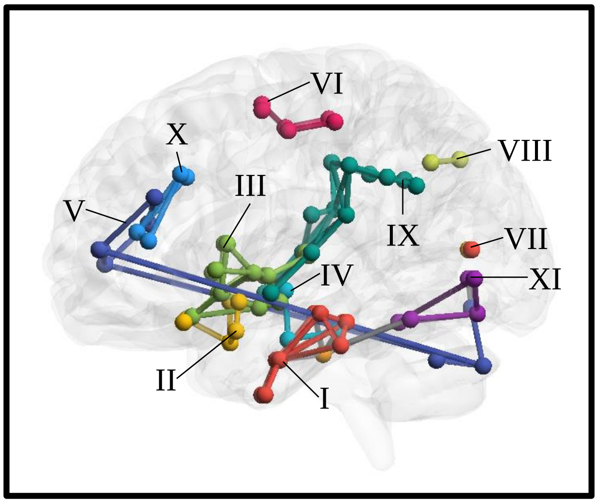

Supplementary Figure 3. The 'core' network of connections which persisted across groups and conditions, derived as depicted in Supplementary Figure 2. Colors represent discrete subgraphs elicited via Louvain community detection algorithm (see Figure 5 of the main text). These subgraphs are labeled in the inset panel on the left. ROI abbreviations are defined in Supplementary Table 1.

Supplementary Figure 4 ***(next page).*** Nodal communities derived from control participants using the Louvain community detection algorithm, examining cuff-deflated and cuff-inflated conditions separately. Pons, brainstem, and midbrain; hippocampus and parahippocampal cortex; anterior cingulate and medial prefrontal cortex; cerebellum and dorsolateral prefrontal cortex; basal ganglia, nucleus accumbens, and hypothalamus; amygdala and entorhinal cortex; periaqueductal grey and dorsal posterior insula; and supramarginal and angular gyri were assigned to the same communities in both conditions. Also note the relative sparsity of connections compared to TMD participants (Supplementary Figure 5).

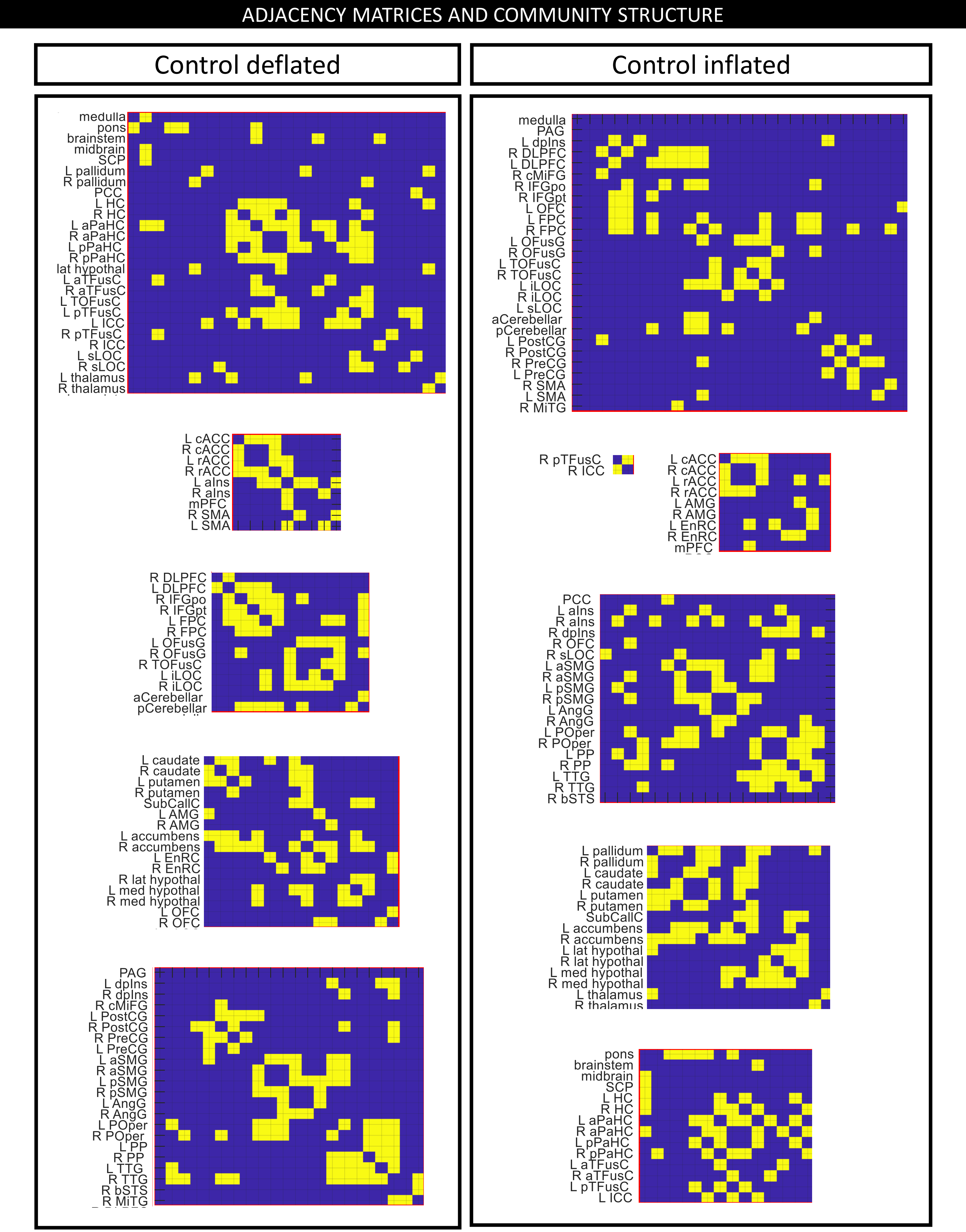
ADJACENCY MATRICES AND COMMUNITY STRUCTURE
Control deflated
Control inflated
medulla
pons
brainstem
midbrain
SCP
L pallidum
R pallidum
PCC
L HC
R HC
L aPaHC
R aPaHC
L pPaHC
R pPaHC
lat hypothal
L aTFusC
R aTFusC
L TOFusC
L pTFusC
L ICC
R pTFusC
R ICC
L sLOC
R sLOC
L thalamus
R thalamus
L cACC
R cACC
L rACC
R rACC
L aIns
R aIns
mPFC
R SMA
L SMA
R DLPFC
L DLPFC
R IFGpo
R IFGpt
L FPC
R FPC
L OFusG
R OFusG
R TOFusC
L iLOC
R iLOC
aCerebellar
pCerebellar
L caudate
R caudate
L putamen
R putamen
SubCallC
L AMG
R AMG
L accumbens
R accumbens
L EnRC
R EnRC
R lat hypothal
L med hypothal
R med hypothal
L OFC
R OFC
PAG
L dpIns
R dpIns
R cMiFG
L PostCG
R PostCG
R PreCG
L PreCG
L aSMG
R aSMG
L pSMG
R pSMG
L AngG
R AngG
L POper
R POper
L PP
R PP
L TTG
R TTG
R bSTS
R MiTG
medulla
PAG
L dpIns
R DLPFC
L DLPFC
R cMiFG
R IFGpo
R IFGpt
L OFC
L FPC
R FPC
L OFusG
R OFusG
L TOFusC
R TOFusC
L iLOC
R iLOC
L sLOC
aCerebellar
pCerebellar
L PostCG
R PostCG
R PreCG
L PreCG
R SMA
L SMA
R MiTG
R pTFusC
R ICC
L cACC
R cACC
L rACC
R rACC
L AMG
R AMG
L EnRC
R EnRC
mPFC
PCC
L aIns
R aIns
R dpIns
R OFC
R sLOC
L aSMG
R aSMG
L pSMG
R pSMG
L AngG
R AngG
L POper
R POper
L PP
R PP
L TTG
R TTG
R bSTS
L pallidum
R pallidum
L caudate
R caudate
L putamen
R putamen
SubCallC
L accumbens
R accumbens
L lat hypothal
R lat hypothal
L med hypothal
R med hypothal
L thalamus
R thalamus
pons
brainstem
midbrain
SCP
L HC
R HC
L aPaHC
R aPaHC
L pPaHC
R pPaHC
L aTFusC
R aTFusC
L pTFusC
L ICC

Supplementary Figure 5 ***(next page).*** Nodal communities derived from TMD participants using the Louvain community detection algorithm, examining cuff-deflated and cuff-inflated conditions separately. Communities were largely conserved in these participants across the cuff-deflated and cuff-inflated conditions, with the notable exception of dorsolateral prefrontal cortex (DLPFC), anterior cingulate (ACC), and medial prefrontal cortex (mPFC), and more connections were present in both conditions compared to control participants (compare Supplementary Figure 4).

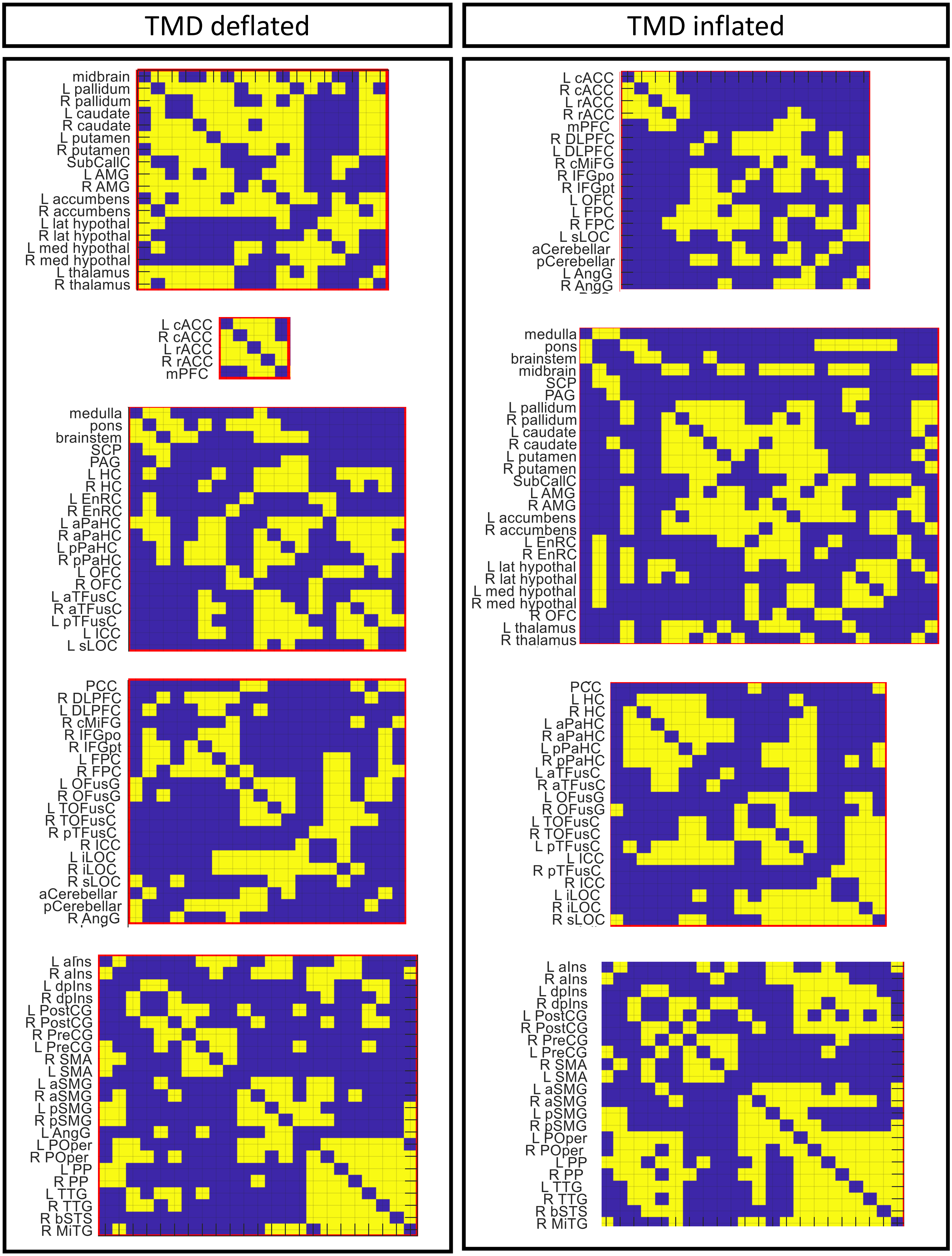
TMD deflated
TMD inflated
midbrain
L pallidum
R pallidum
L caudate
R caudate
L putamen
R putamen
SubCallC
L AMG
R AMG
L accumbens
R accumbens
L lat hypothal
R lat hypothal
L med hypothal
R med hypothal
L thalamus
R thalamus
L cACC
R cACC
L rACC
R rACC
mPFC
medulla
pons
brainstem
SCP
PAG
L HC
R HC
L EnRC
R EnRC
L aPaHC
R aPaHC
L pPaHC
R pPaHC
L OFC
R OFC
L aTFusC
R aTFusC
L pTFusC
L ICC
L sLOC
PCC
R DLPFC
L DLPFC
R cMiFG
R IFGpo
R IFGpt
L FPC
R FPC
L OFusG
R OFusG
L TOFusC
R TOFusC
R pTFusC
R ICC
L iLOC
R iLOC
R sLOC
aCerebellar
pCerebellar
R AngG
L aIns
R aIns
L dpIns
R dpIns
L PostCG
R PostCG
R PreCG
L PreCG
R SMA
L SMA
L aSMG
R aSMG
L pSMG
R pSMG
L AngG
L POper
R POper
L PP
R PP
L TTG
R TTG
R bSTS
R MiTG
L cACC
R cACC
L rACC
R rACC
mPFC
R DLPFC
L DLPFC
R cMiFG
R IFGpo
R IFGpt
L OFC
L FPC
R FPC
L sLOC
aCerebellar
pCerebellar
L AngG
R AngG
medulla
pons
brainstem
midbrain
SCP
PAG
L pallidum
R pallidum
L caudate
R caudate
L putamen
R putamen
SubCallC
L AMG
R AMG
L accumbens
R accumbens
L EnRC
R EnRC
L lat hypothal
R lat hypothal
L med hypothal
R med hypothal
R OFC
L thalamus
R thalamus
PCC
L HC
R HC
L aPaHC
R aPaHC
L pPaHC
R pPaHC
L aTFusC
R aTFusC
L OFusG
R OFusG
L TOFusC
R TOFusC
L pTFusC
L ICC
R pTFusC
R ICC
L iLOC
R iLOC
R sLOC
L aIns
R aIns
L dpIns
R dpIns
L PostCG
R PostCG
R PreCG
L PreCG
R SMA
L SMA
L aSMG
R aSMG
L pSMG
R pSMG
L POper
R POper
L PP
R PP
L TTG
R TTG
R bSTS
R MiTG

Supplementary Figure 6 ***(next page).*** Nodal degree (panel *A*) and betweenness centrality (panel *B*, normalized to the total number of node pairs) for the two groups and conditions. Each group × condition is represented in a separate column in both panels: *DEFL,* deflated; *INFL,* inflated. Brighter colors represent a higher degree of connectivity or ‘importance/hubness’ (centrality). Note that nodal degree is higher for TMD participants, relative to controls, across both conditions, indicating that TMD ROIs are more likely to be correlated with each other, and thus TMD participants are observed to ‘have more connections’ or ‘are over-wired.’ Conversely, betweenness centrality is lower in TMD participants relative to controls, indicating a loss of ‘hub-ness,’ decreased ‘node importance,’ or ‘hub disruption.’ (Compare Supplementary Figure 7 and Figure 6 in the main text.)

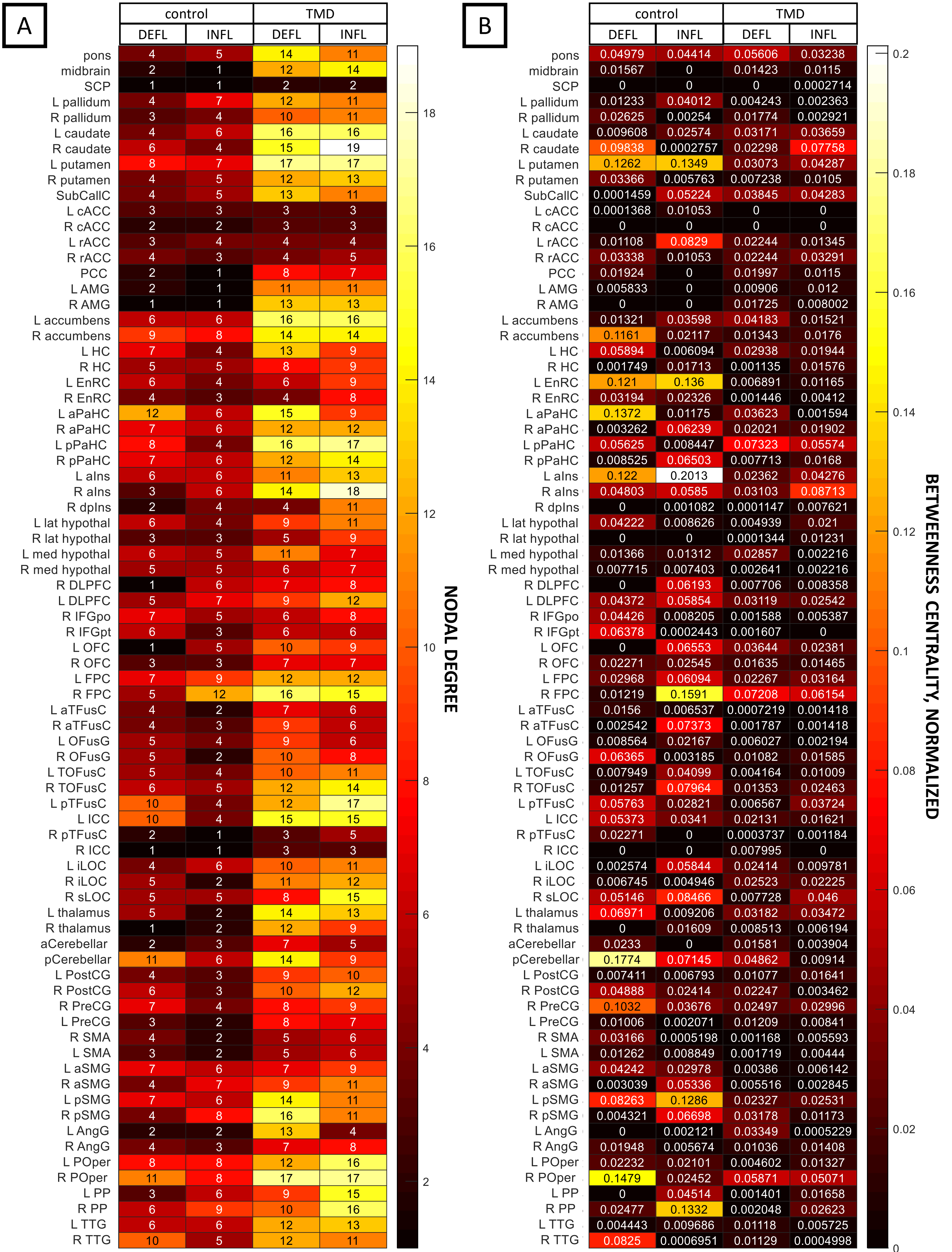

A	control		TMD	
	DEFL	INFL	DEFL	INFL
pons	4	5	14	11
midbrain	2	1	12	14
SCP	1	1	2	2
L pallidum	4	7	12	11
R pallidum	3	4	10	11
L caudate	4	6	16	16
R caudate	6	4	15	19
L putamen	8	7	17	17
R putamen	4	5	12	13
SubCallC	4	5	13	11
L cACC	3	3	3	3
R cACC	2	2	3	3
L rACC	3	4	4	4
R rACC	4	3	4	5
PCC	2	1	8	7
L AMG	2	1	11	11
R AMG	1	1	13	13
L accumbens	6	6	16	16
R accumbens	9	8	14	14
L HC	7	4	13	9
R HC	5	5	8	9
L EnRC	6	4	6	9
R EnRC	4	3	4	8
L aPaHC	12	6	15	9
R aPaHC	7	6	12	12
L pPaHC	8	4	16	17
R pPaHC	7	6	12	14
L aIns	6	6	11	13
R aIns	3	6	14	18
R dpIns	2	4	4	11
L lat hypothal	6	4	9	11
R lat hypothal	3	3	5	9
L med hypothal	6	5	11	7
R med hypothal	5	5	6	7
R DLPFC	1	6	7	8
L DLPFC	5	7	9	12
R IFGpo	7	5	6	8
R IFGpt	6	3	6	6
L OFC	1	5	10	9
R OFC	3	3	7	7
L FPC	7	9	12	12
R FPC	5	12	16	15
L aTFusC	4	2	7	6
R aTFusC	4	3	9	6
L OFusG	5	4	9	6
R OFusG	5	2	10	8
L TOFusC	5	4	10	11
R TOFusC	6	5	12	14
L pTFusC	10	4	12	17
L ICC	10	4	15	15
R pTFusC	2	1	3	5
R ICC	1	1	3	3
L iLOC	4	6	10	11
R iLOC	5	2	11	12
R sLOC	5	5	8	15
L thalamus	5	2	14	13
R thalamus	1	2	12	9
aCerebellar	2	3	7	5
pCerebellar	11	6	14	9
L PostCG	4	3	9	10
R PostCG	6	3	10	12
R PreCG	7	4	8	9
L PreCG	3	2	8	7
R SMA	4	2	5	6
L SMA	3	2	5	6
L aSMG	7	6	7	9
R aSMG	4	7	9	11
L pSMG	7	6	14	11
R pSMG	4	8	16	11
L AngG	2	2	13	4
R AngG	4	3	7	8
L POper	8	8	12	16
R POper	11	8	17	17
L PP	3	6	9	15
R PP	6	9	10	16
L TTG	6	6	12	13
R TTG	10	5	12	11

B	control		TMD	
	DEFL	INFL	DEFL	INFL
pons	0.04979	0.04414	0.05606	0.03238
midbrain	0.01567	0	0.01423	0.0115
SCP	0	0	0	0.0002714
L pallidum	0.01233	0.04012	0.004243	0.002363
R pallidum	0.02625	0.00254	0.01774	0.002921
L caudate	0.009608	0.02574	0.03171	0.03659
R caudate	0.09838	0.0002757	0.02298	0.07758
L putamen	0.1262	0.1349	0.03073	0.04287
R putamen	0.03366	0.005763	0.007238	0.0105
SubCallC	0.0001459	0.05224	0.03845	0.04283
L cACC	0.0001368	0.01053	0	0
R cACC	0	0	0	0
L rACC	0.01108	0.0829	0.02244	0.01345
R rACC	0.03338	0.01053	0.02244	0.03291
PCC	0.01924	0	0.01997	0.0115
L AMG	0.005833	0	0.00906	0.012
R AMG	0	0	0.01725	0.008002
L accumbens	0.01321	0.03598	0.04183	0.01521
R accumbens	0.1161	0.02117	0.01343	0.0176
L HC	0.05894	0.006094	0.02938	0.01944
R HC	0.001749	0.01713	0.001135	0.01576
L EnRC	0.121	0.136	0.006891	0.01165
R EnRC	0.03194	0.02326	0.001446	0.00412
L aPaHC	0.1372	0.01175	0.03623	0.001594
R aPaHC	0.003262	0.06239	0.02021	0.01902
L pPaHC	0.05625	0.008447	0.07323	0.05574
R pPaHC	0.008525	0.06503	0.007713	0.0168
L aIns	0.122	0.2013	0.02362	0.04276
R aIns	0.04803	0.0585	0.03103	0.08713
R dpIns	0	0.001082	0.0001147	0.007621
L lat hypothal	0.04222	0.008626	0.004939	0.021
R lat hypothal	0	0	0.0001344	0.01231
L med hypothal	0.01366	0.01312	0.02857	0.002216
R med hypothal	0.007715	0.007403	0.002641	0.002216
R DLPFC	0	0.06193	0.007706	0.008358
L DLPFC	0.04372	0.05854	0.03119	0.02542
R IFGpo	0.04426	0.008205	0.001588	0.005387
R IFGpt	0.06378	0.0002443	0.001607	0
L OFC	0	0.06553	0.03644	0.02381
R OFC	0.02271	0.02545	0.01635	0.01465
L FPC	0.02968	0.06094	0.02267	0.03164
R FPC	0.01219	0.1591	0.07208	0.06154
L aTFusC	0.0156	0.006537	0.0007219	0.001418
R aTFusC	0.002542	0.07373	0.001787	0.001418
L OFusG	0.008564	0.02167	0.006027	0.002194
R OFusG	0.06365	0.003185	0.01082	0.01585
L TOFusC	0.007949	0.04099	0.004164	0.01009
R TOFusC	0.01257	0.07964	0.01353	0.02463
L pTFusC	0.05763	0.02821	0.006567	0.03724
L ICC	0.05373	0.0341	0.02131	0.01621
R pTFusC	0.02271	0	0.0003737	0.001184
R ICC	0	0	0.007995	0
L iLOC	0.002574	0.05844	0.02414	0.009781
R iLOC	0.006745	0.004946	0.02523	0.02225
R sLOC	0.05146	0.08466	0.007728	0.046
L thalamus	0.06971	0.009206	0.03182	0.03472
R thalamus	0	0.01609	0.008513	0.006194
aCerebellar	0.0233	0	0.01581	0.003904
pCerebellar	0.1774	0.07145	0.04862	0.00914
L PostCG	0.007411	0.006793	0.01077	0.01641
R PostCG	0.04888	0.02414	0.02247	0.003462
R PreCG	0.1032	0.03676	0.02497	0.02996
L PreCG	0.01006	0.002071	0.01209	0.00841
R SMA	0.03166	0.0005198	0.001168	0.005593
L SMA	0.01262	0.008849	0.001719	0.00444
L aSMG	0.04242	0.02978	0.00386	0.006142
R aSMG	0.003039	0.05336	0.005516	0.002845
L pSMG	0.08263	0.1286	0.02327	0.02531
R pSMG	0.004321	0.06698	0.03178	0.01173
L AngG	0	0.002121	0.03349	0.0005229
R AngG	0.01948	0.005674	0.01036	0.01408
L POper	0.02232	0.02101	0.004602	0.01327
R POper	0.1479	0.02452	0.05871	0.05071
L PP	0	0.04514	0.001401	0.01658
R PP	0.02477	0.1332	0.002048	0.02623
L TTG	0.004443	0.009686	0.01118	0.005725
R TTG	0.0825	0.0006951	0.01129	0.0004998

A CONTROL PARTICIPANTS

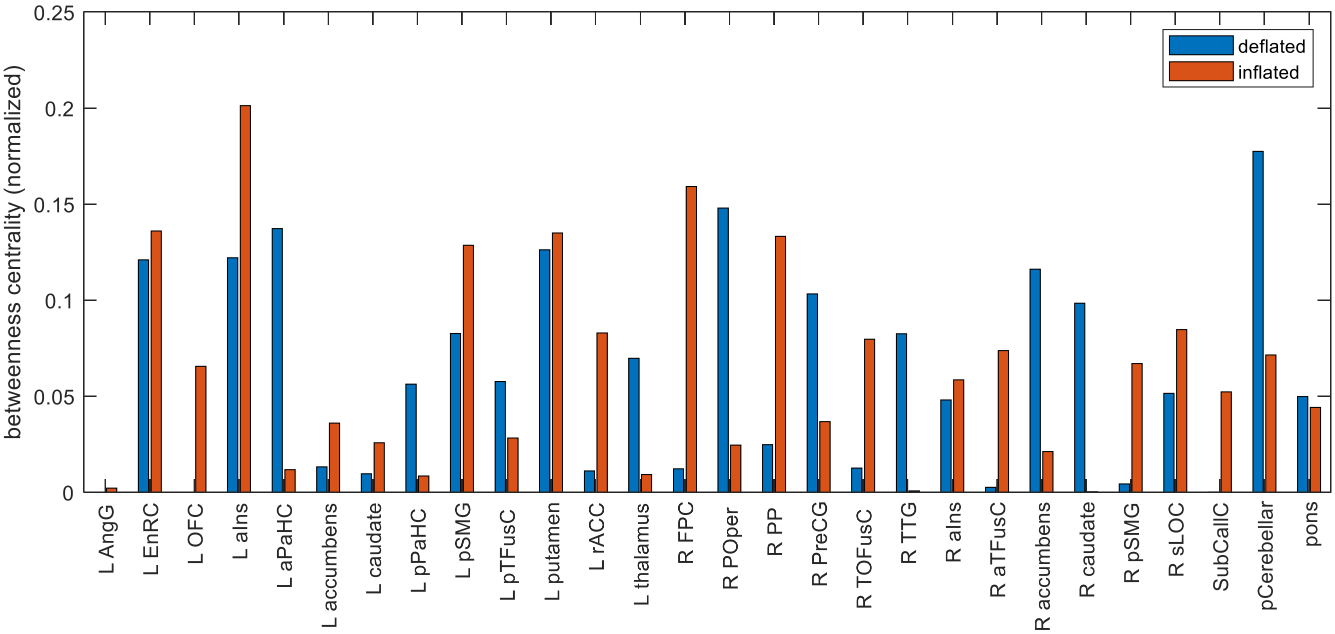

B TMD PARTICIPANTS

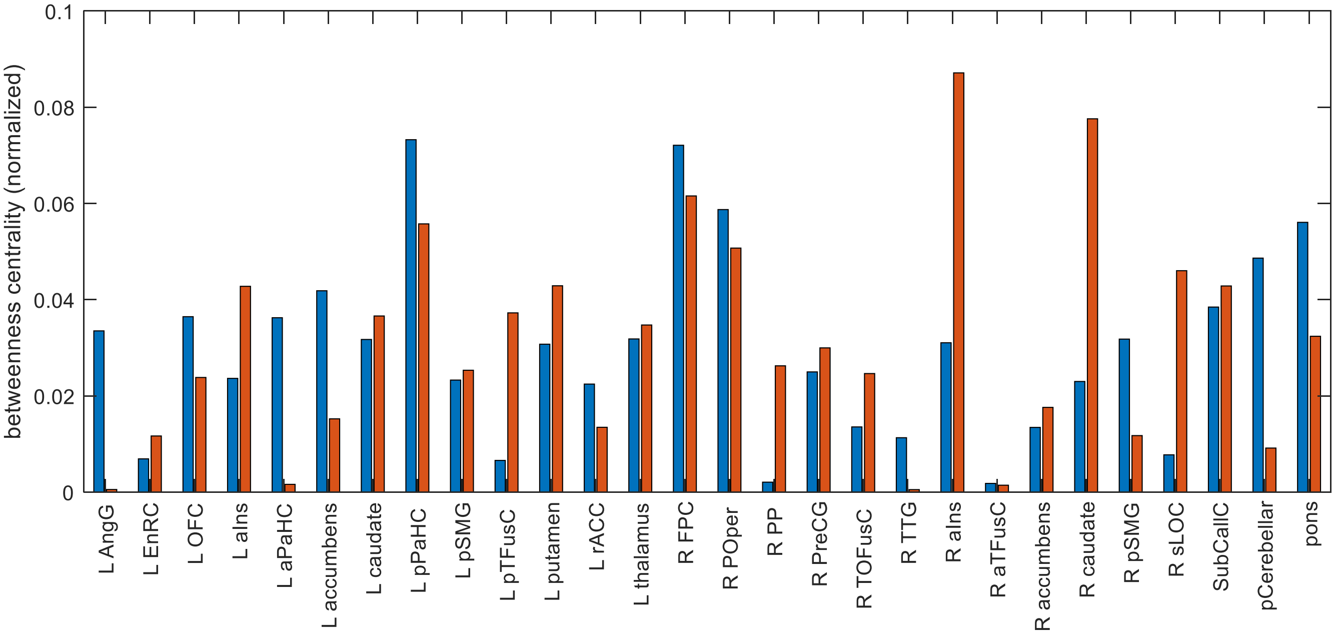

Supplementary Figure 7. Betweenness centrality ('hub-ness,' normalized to the total number of nodal pairs) for (*A*) control and (*B*) TMD participants. Centralities for the cuff-deflated condition are shown in blue, and for the cuff-inflated condition in orange. Also note that the y-axis scaling differs between panels (*A*) and (*B*), in order to more clearly show small centralities in each group and condition.

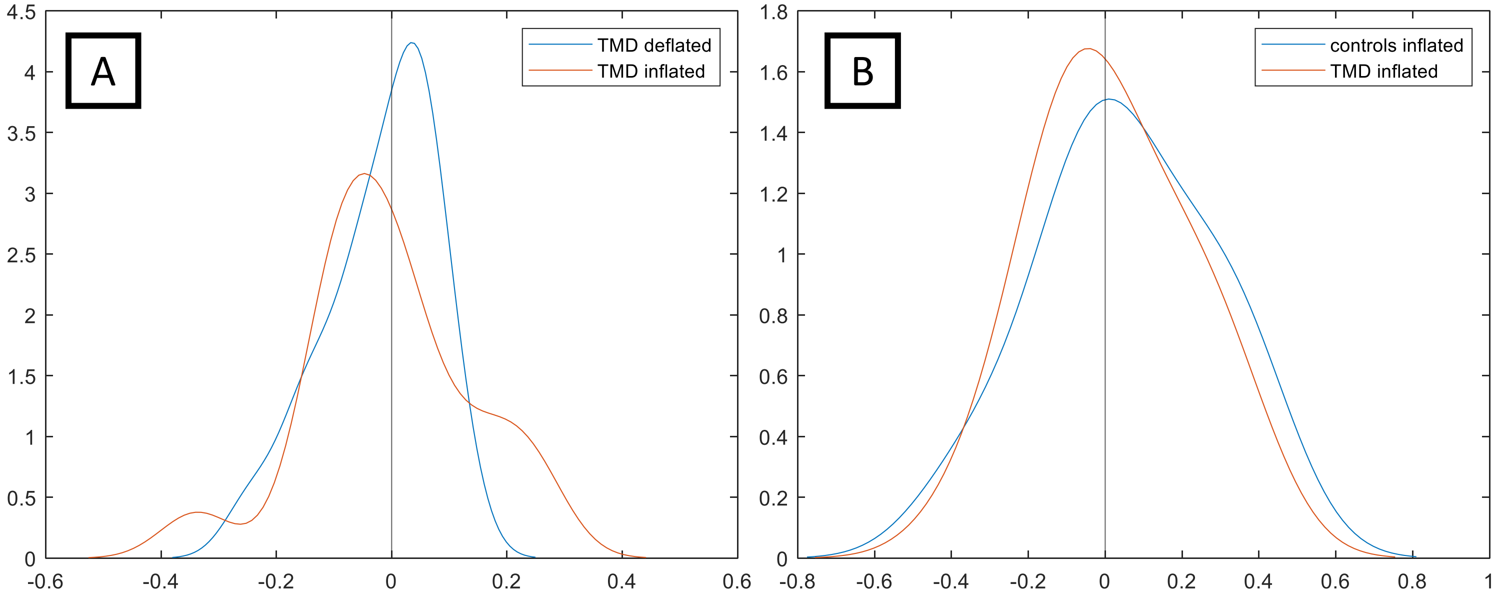

Supplementary Figure 8. (*A*) Kernel density plot of normalized connectivity values for the left thalamus-to-right orbitofrontal cortex connection in TMD participants in the deflated (blue) and inflated (orange) condition. This connection was indicated to differ between conditions within the TMD subjects following thresholding with NBS statistics. As shown in the density plot, both control and TMD participants' connectivities for this connection diverged from zero and differed substantively in mean to yield a significant difference in the two-sample t-test. (*B*) Kernel density plot of normalized connectivity values for the left parietal operculum-to-left dorsal posterior insula connection in control (blue) and TMD (orange) participants in the inflated condition. This connection was indicated as 'present' in the TMD-inflated, but not control-inflated, adjacency matrix. The density plot indicates that this finding was due to skewness in TMD participants' connectivity values, bringing the mean to the left of zero and yielding a significant difference in the one-sample t-tests used to generate the adjacency matrix.

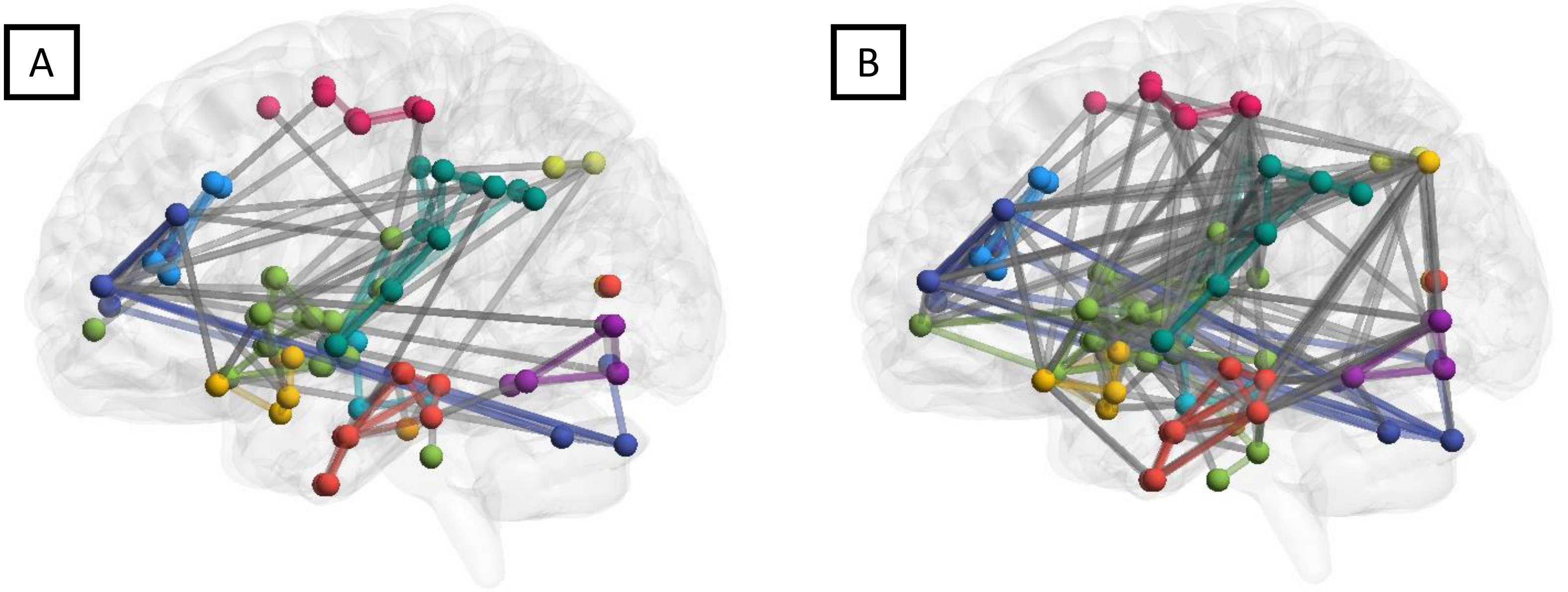

Supplementary Figure 9. Depiction of the overall functional connectivity of *(A*) control and *(B*) TMD participants in the cuff-inflated condition. Note that TMDs exhibit a greater number of nodal connections in this condition compared to controls. Colors represent nodal community, as determined with the Louvain community detection algorithm and shown in Supplementary Figure 3 and Figure 5 of the main text.
